# A multiscale brain network model links Alzheimer’s disease-mediated neuronal hyperactivity to large-scale oscillatory slowing

**DOI:** 10.1186/s13195-022-01041-4

**Published:** 2022-07-25

**Authors:** Anne M. van Nifterick, Alida A. Gouw, Ronald E. van Kesteren, Philip Scheltens, Cornelis J. Stam, Willem de Haan

**Affiliations:** 1 Alzheimer Center Amsterdam, Neurology, Vrije Universiteit Amsterdam, Amsterdam UMC location VUmc, Amsterdam, The Netherlands; 2 Clinical Neurophysiology and MEG Center, Neurology, Vrije Universiteit Amsterdam, Amsterdam UMC location VUmc, Amsterdam, The Netherlands; 3 Amsterdam Neuroscience, Neurodegeneration, Amsterdam, The Netherlands; 4 Department of Molecular and Cellular Neurobiology, Center for Neurogenomics and Cognitive Research, Vrije Universiteit Amsterdam, Amsterdam, The Netherlands

**Keywords:** MEG, Alzheimer’s disease, Oscillatory slowing, Hyperexcitability, Neural mass models, Neuronal network, Computational modeling

## Abstract

**Background:**

Neuronal hyperexcitability and inhibitory interneuron dysfunction are frequently observed in preclinical animal models of Alzheimer’s disease (AD). This study investigates whether these microscale abnormalities explain characteristic large-scale magnetoencephalography (MEG) activity in human early-stage AD patients.

**Methods:**

To simulate spontaneous electrophysiological activity, we used a whole-brain computational network model comprised of 78 neural masses coupled according to human structural brain topology. We modified relevant model parameters to simulate six literature-based cellular scenarios of AD and compare them to one healthy and six contrast (non-AD-like) scenarios. The parameters include excitability, postsynaptic potentials, and coupling strength of excitatory and inhibitory neuronal populations. Whole-brain spike density and spectral power analyses of the simulated data reveal mechanisms of neuronal hyperactivity that lead to oscillatory changes similar to those observed in MEG data of 18 human prodromal AD patients compared to 18 age-matched subjects with subjective cognitive decline.

**Results:**

All but one of the AD-like scenarios showed higher spike density levels, and all but one of these scenarios had a lower peak frequency, higher spectral power in slower (theta, 4–8Hz) frequencies, and greater total power. Non-AD-like scenarios showed opposite patterns mainly, including reduced spike density and faster oscillatory activity. Human AD patients showed oscillatory slowing (i.e., higher relative power in the theta band mainly), a trend for lower peak frequency and higher total power compared to controls. Combining model and human data, the findings indicate that neuronal hyperactivity can lead to oscillatory slowing, likely due to hyperexcitation (by hyperexcitability of pyramidal neurons or greater long-range excitatory coupling) and/or disinhibition (by reduced excitability of inhibitory interneurons or weaker local inhibitory coupling strength) in early AD.

**Conclusions:**

Using a computational brain network model, we link findings from different scales and models and support the hypothesis of early-stage neuronal hyperactivity underlying E/I imbalance and whole-brain network dysfunction in prodromal AD.

**Supplementary Information:**

The online version contains supplementary material available at 10.1186/s13195-022-01041-4.

## Background

Evidence for neuronal hyperactivity and network hyperexcitability in the early trajectory of Alzheimer’s disease (AD), as well as its role in memory dysfunction, is accumulating from multiple lines of study [9–11, 16, 20, 58, 64, 92, 98, 99]. In AD animal models, in vivo calcium imaging have revealed hyperactive neurons in the hippocampus even before substantial amyloid-β plaque load [9, 10], and electroencephalography (EEG) recordings have showed cortical and hippocampal epileptiform spikes [53, 58]. In humans, functional magnetic resonance imaging (fMRI) studies provided support for the early stage neuronal hyperactivity hypothesis, showing task-based hyperactivation of the default mode network and hippocampus in asymptomatic and mildly impaired AD subjects [43, 64, 65]. Hippocampal hyperactivation and cognitive deficits in amnestic mild cognitively impaired (MCI) patients could be partially rescued by *levetiracetam* treatment [4, 5]. Though fMRI indicates a relative change in oxygenated blood levels and is not directly measuring neuronal activity, EEG or magnetoencephalography (MEG) recordings capture neuronal activity directly (on a group level). EEG/MEG studies have provided additional support for an early stage hyperexcitable network in AD, by showing that humans at risk of developing AD dementia also have a higher risk of EEG activity patterns associated with epilepsy [26, 40, 93, 95]. It remains uncertain, however, how microscale neuronal hyperactivity (defined in this study as higher absolute spike density of the excitatory pyramidal neurons) translates into macroscale features as determined with noninvasive and direct measures of neuronal network activity such as EEG or MEG. Understanding how microscale neuronal hyperactivity is observed from large-scale electromagnetic signals is of significant importance as it allows early detection of AD-related neuronal dysfunction and is a potential therapeutic target: restoring neuronal imbalance may prevent irreversible neuronal degeneration, network disconnection, and cognitive decline [70].

EEG/MEG renders a unique opportunity to assess how excitation-inhibition (E/I) balance, i.e., the ratio of excitatory and inhibitory neuronal firing rates in a network, is perturbed in disease. It captures the electromagnetic field produced by continuously changing synaptic currents in many postsynaptic pyramidal neurons with regular orientation perpendicular to the cortical surface and, by source modeling, MEG even allows detection of synaptic activity in the deeper, subcortical, brain regions including the hippocampus [3, 25]. Synaptic dysfunction and E/I imbalance in smaller assemblies of interconnected neurons may cause abnormal network activity (i.e., larger interconnected circuits involving different brain regions) and influences the shape and behavior of neural oscillations important for cognitive function [12, 81]. Although EEG/MEG signals, in theory, hold information about desynchronized neuronal activity and E/I imbalance, the translation of local neuronal firing to whole-brain activity introduces a major increase in complexity and requires additional research.

**Fig. 1:**
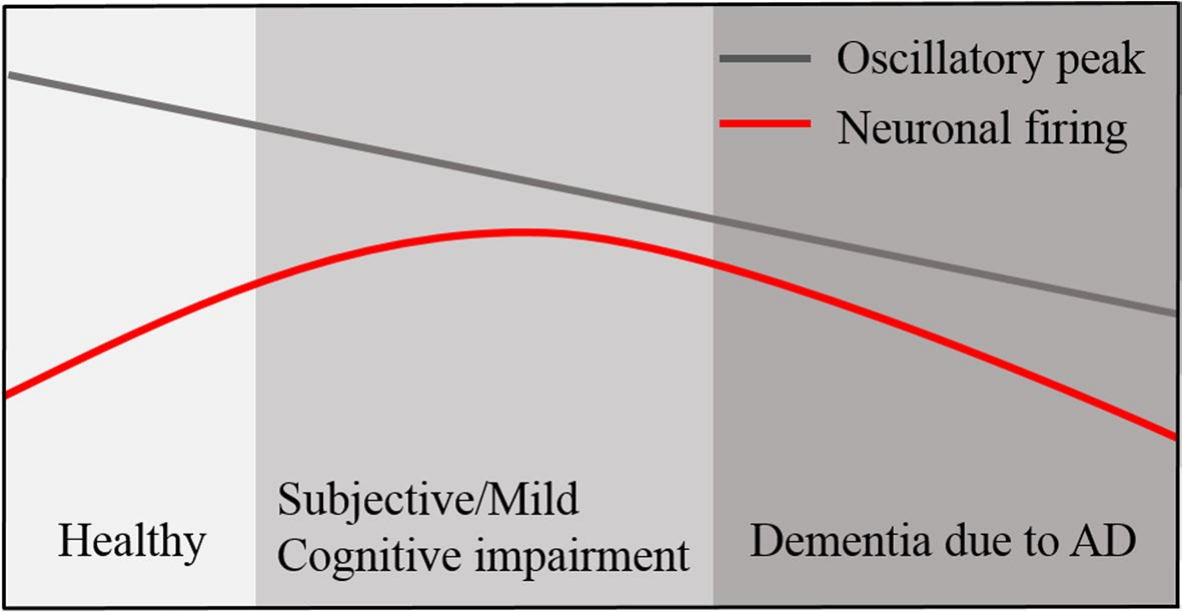
Illustration of the hypothesized course of peak frequency and neuronal activity across different stages of AD. This figure presents a working model based on previous results from electrophysiological studies. While the oscillatory peak frequency is progressively slowing over time, neuronal activity is hypothesized to follow an inverted U-shape curve across the course of AD. Please note that the group with subjective cognitive complaints presented here refers to subjects with confirmed amyloid pathology (by CSF or PET), in contrast to subjects with a diagnosis of subjective cognitive decline without amyloid pathology that are included as elderly healthy controls in this study

A robust macroscale electromagnetic finding in patients across the AD continuum is a gradual diffuse and progressive slowing of the oscillatory cortical rhythms, in particular higher relative theta power and lower beta power, followed in later stages by a decrease in alpha power and increase in delta power as well as a lower peak frequency (i.e., the dominant frequency between 4 and 13 Hz) (Fig. 1) [17, 24, 25]. Intuitively, oscillatory slowing likely reflects a gradual decrease of neuronal activity due to, for instance, amyloid-β accumulation and subsequent neurodegeneration, although numerous studies now point towards the contrary. Possibly, AD progression is initially characterized by neuronal hyperactivity associated with amyloid-β pathology, followed by hypoactivity and neuronal loss through excitotoxicity and pathological tau proteins in later disease stages, and thus may follow a biphasic course (Fig. 1) [8, 16, 63, 68, 75]. Several functional connectivity studies have examined the temporal correlation in neurophysiological signals between distinct brain regions and provided support for this biphasic theory by showing hypersynchronization in the early stages of AD and a global disconnected network in more late stages [16, 48, 56, 57, 63, 75, 77]. Results from optogenetic stimulation studies in animal models show a link between specific neuronal firing rates and modulation of oscillatory power and indicate a positive relation between inhibitory interneuron firing rate and high frequency (gamma) oscillatory power, as well as between pyramidal cell activity and power of low-frequency oscillations [13, 74]. Whether such a causal relationship between neuronal activity and oscillatory power also exists in humans, and whether neuronal hyperactivity relates to slowing of the oscillatory activity in particular, has not been elucidated yet.

**Fig. 2:**
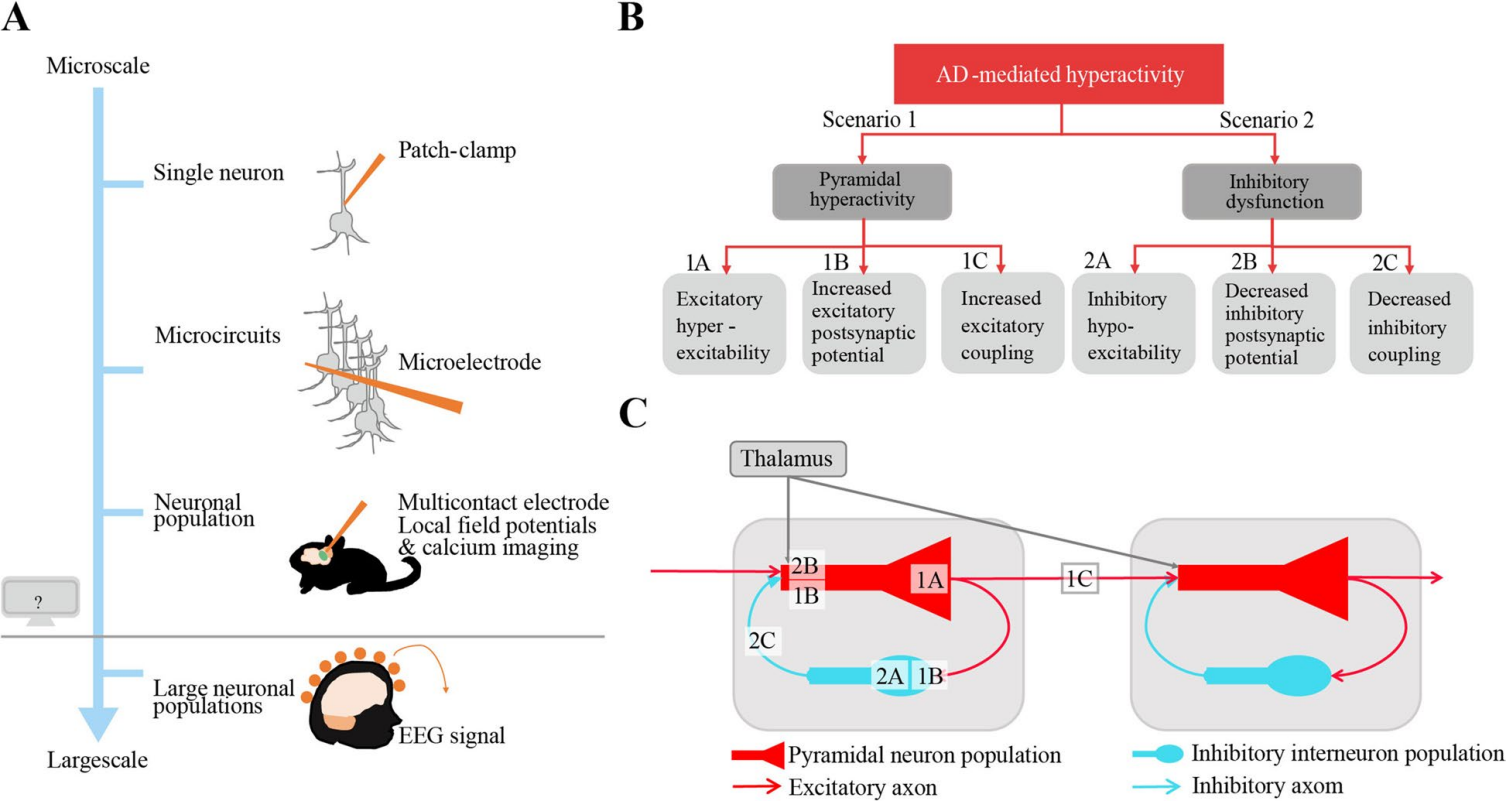
AD-mediated microscale neuronal hyperactivity and its translation to large-scale EEG/MEG signals. **A** By using a computational brain network model, we can implement empirically informed AD neuronal pathology as measured by single cell patch-clamp recordings, microcircuit local field potentials, or calcium imaging, and investigate the effects on macroscale brain oscillations as measured by whole-brain EEG/MEG in human prodromal AD patients. **B** Schematic illustration of the different scenarios of AD-mediated changes in neuronal activity implemented in the model. Scenario 1: pyramidal neuronal hyperactivity by simulation of A: (intrinsically) increased excitability of excitatory neurons, or by B: increased excitatory postsynaptic potentials of pyramidal (and inhibitory) neurons, or by C: increased excitatory to excitatory coupling strength. Scenario 2: inhibitory neuronal dysfunction by simulation of A: (intrinsically) decreased excitability of inhibitory neurons, or by B: decreased inhibitory postsynaptic potential in the excitatory population, or by C: decreased inhibitory to excitatory synaptic coupling. **C** Simplified presentation of the computational dynamic brain network model used in this study. Only two coupled neural masses are shown for simplicity. The number-letter combinations correspond to the different AD-like scenarios in B and their location reflects the virtual spatial location. For more details about the scenarios, we refer to Table 2

Ideally, a direct translation to identify the cellular mechanisms underlying large-scale changes in network activity is made by simultaneous invasive intracranial electrode recordings and non-invasive whole-brain EEG/MEG in human AD patients [49]. This type of dual recording is considered unethical, and, therefore, computational dynamic brain network modeling is particularly useful to implement AD-driven pathophysiology on a local level and study its effect on large-scale network activity. De Haan et al. have previously simulated AD-like network disruption with a simple rule of activity-dependent degeneration (i.e*.*, the cortical regions with the highest activity levels developed structural connectivity loss) that resulted in multiple AD-like electromagnetic network signatures including oscillatory slowing, higher spectral power, disruption of functional network topology, and hub vulnerability [16]. Interestingly, upon activity-dependent degeneration, the model output showed an initial rise in spike density of the excitatory neurons before a collapse [16], indicating potential early stage neuronal hyperactivity in AD. More recently, a computational network model study with AD-dependent local disinhibition (and thus hyperexcitation) has showed higher power in the theta frequency band, providing a possible pathophysiological mechanism of oscillatory slowing [80]. These findings indicate that dynamic network modeling can assist in bridging scales of neuronal activity and our understanding of pathophysiology as observed in AD (Fig. 2A )[30].

What are then the cell-physiological mechanisms of neuronal hyperactivity that could explain aberrant oscillatory network activity in early AD? Although many studies suggest amyloid-β-induced pyramidal neuron hyperexcitability [54], either indirectly by observing increased neuronal spiking in cortical and hippocampal brain regions [9–11, 68, 99] and spontaneous epileptiform activity in transgenic animal models of AD [53, 58–60, 67], or directly through electrophysiological recordings in vivo [47] and in brain slices derived from transgenic mice [53, 82], others showed impairments of inhibitory interneurons as a mechanism of network hyperexcitability in AD [2]. In particular, functional impairments of fast-spiking parvalbumin positive interneurons [15, 38, 52, 61, 92], but also of somatostatin positive interneurons [71], have been demonstrated. In addition, amyloid-β interferes with the number of postsynaptic inhibitory receptors (on pyramidal neurons) [88] and excessively activates inhibitory receptors on the inhibitory neuron population itself, leading to disinhibition of the excitatory neurons [36, 66]. Furthermore, a loss of functional inhibitory synapses in AD has been suggested as important contributor of aberrant neuronal network activity [21, 28, 73, 83]. Finally, amyloid-β may introduce E/I imbalance indirectly through blocking synaptically released glutamate reuptake by astrocytes [50, 99] elevating levels of glutamate in the synaptic cleft and thus initially increasing postsynaptic excitatory currents, but ultimately inducing synaptic depression and reducing the peak amplitudes of the excitatory currents (for review [96]).

In this study, we use a computational brain network model to examine the cell physiological basis of characteristic global spectral power changes in early stages of AD. We evaluate the compatibility of AD-driven mechanisms of neuronal hyperactivity with large-scale oscillatory slowing (Fig. 2B). Based on studies of AD animal and cell models, we consider the following scenarios of AD-like neuronal dysfunction: pyramidal neuronal hyperactivity by (intrinsic) hyperexcitability of pyramidal neurons (1A), disturbed glutamate homeostasis simulated by increased excitatory postsynaptic potentials (1B), and increased long-range excitatory to excitatory coupling (1C). Inhibitory neuronal dysfunction by (intrinsic) inhibitory hypoexcitability (2A), through decreased inhibitory post-synaptic potential in pyramidal neurons (2B), and by decreased inhibitory to excitatory coupling strength (2C). By generating spectral activity profiles for each scenario, we can assess its compatibility with empirical MEG findings of early stage AD patients.

## Methods

### Computational modeling

#### The coupled neural mass model

The computational model was comprised of 78 single neural mass models (NMMs) that were each considered a cortical patch and represented the average activity of a large population of interconnected excitatory and inhibitory neurons (Fig. 2C) [51, 97]. The fluctuation of the average membrane potential of the main pyramidal neuronal populations mimicked the EEG or MEG signals. Besides the multichannel output of fluctuations in average membrane potentials, the model offered a direct readout of neuronal activity by the pulse density output, i.e., the number of excitatory cells firing per unit time. The NMM and its parameters have been informed by histological and biophysical studies and have been originally designed to realistically simulate and explain complex electrophysiological dynamics in the alpha frequency band observed with EEG or MEG [41, 51, 79, 97]). In addition to physiological alpha rhythms (with harmonics extending to higher frequencies), the model has generated EEG phenomena after parameter modification such as seen in patients with encephalopathy or epilepsy [79, 89]. Of note, the model-generated output did not cover the entire human frequency spectrum, although this has been previously attempted by others [19]. We were primarily interested in effects on the dominant alpha rhythm as empirical hallmark in neurophysiological AD data, so we preferred the specificity and relative simplicity of this model. Also when Deco and colleagues assumed a single oscillator in their model, the best fit to empirical MEG data was obtained using a fundamental frequency in the alpha range, further justifying our model choice [19]. To introduce anatomical information and long-range network activity in a healthy brain, we realistically coupled the neural mass models according to a diffusion tensor imaging (DTI)-derived structural connectivity matrix of the human cortex [31]. Coupling between NMMs, if present, was always reciprocal and excitatory. The global coupling factor (S) determined the overall coupling strength between neural masses and was set as default to 1.5 to obtain a network with physiologically plausible oscillations as proposed in previous studies [16, 18]*.* The relevant parameters of the model are listed in Table 1 and based on previous work by de Haan et al. [16]. The NMM has been employed extensively in previous studies addressing a variety of questions and disorders, including the study of evoked potentials, pathological brain rhythms, and the transition between normal and epileptic activity. We refer to these studies and the supplementary material for more detailed descriptions about the model ([16, 62, 79, 90]).

**Table 1:** General neural mass model parameters and their descriptions

Parameter	Description	Value (default)
*t*	Sample time	0.002 s
*P*(*t*)	The pulse density of an input signal to the excitatory population	550 spikes/s^−1^
Noise	Random fluctuations around average level of *P*(*t*)	1.0
*A h*_e_(*t*)	Amplitude of the EPSP	1.6 mV
*A h*_i_(*t*)	Amplitude of the IPSP	32 mV
*a h*_e_(*t*)	Shape parameter of EPSP	55 s^−1^
*b h*_e_(*t*)	Shape parameter of EPSP	605 s^−1^
*a h*_i_(*t*)	Shape parameter of IPSP	27.5 s^−1^
*b h*_i_(*t*)	Shape parameter of IPSP	55 s^−1^
*g*	Parameter of sigmoid function that relates membrane potential to impulse density	25 s^−1^
*q*	Parameter of sigmoid function that relates membrane potential to impulse density	0.34 mV^−1^
Vd_1_	Threshold potential used in the sigmoid function that relates membrane potential to impulse density for main population of excitatory neurons	7 mV
Vd_2_	Threshold potential used in sigmoid function that relates membrane potential to impulse density for inhibitory neurons	7 mV
C1	Connection strength between main population of excitatory and inhibitory neurons	32
C2	Connection strength between inhibitory neurons and main population of excitatory neurons	3
*S*	Coupling strength between neural masses (gain factor)	1.5
*T*	Time delay for the coupling between neural masses	0.002 s
*N*	Number of neural masses (/nodes) in network model	78

The defined values represent the values as used in default, i.e., control condition in the current study

#### Simulation of pathophysiology in early AD

We implemented different mechanisms of AD (‘AD-like scenarios’) that potentially explain oscillatory slowing in human AD patients. The mechanisms were based upon previous experimental research and implemented in the model through adjusting single relevant parameters of all NMMs, while keeping other parameters constant. Using the coupled neural mass model, we could not only evaluate the effect of these modifications on network activity but also on activity of a smaller scale (i.e., pyramidal spike density). Besides the AD-like mechanisms, non-AD-like 'contrast scenarios' that involvde the exact opposite mechanisms of each AD-like scenario were investigated to show that large-scale oscillatory slowing is not a trivial outcome. We compared each scenario to a 'control' (or healthy) condition with 'normal' parameter values that have been determined previously by others and that are based on biophysical and histological neuronal properties [51, 62, 78].

Taken the normal parameter values previously identified by others, we in- and decreased the parameter values stepwise of all the nodes in the network to generate AD-like and non-AD-like contrast scenarios and evaluated the model output (S5 Fig). For the purpose of clarity, we showed a single representative value for each scenario, one by which the model generated visually evaluated physiological MEG-like signals with periodic oscillations and no epileptic-like activity (S1 Fig). Furthermore, these values were considered still physiologically relevant because they are close to the original value. Because the neural mass model is a mathematically simplified model of activity over a large group of neurons it allowed activity and dynamics of a neuronal population to be summarized in just a couple of variables. The downside of this approach was that a detailed characterization of single neurons is lost and mapping of experimental data to model parameters is thus approximate. Some model parameters matched neuronal characteristics better than others and were therefore more suitable candidates to introduce literature-based AD-like mechanisms. The tested scenarios were as follows and an overview of the modeled AD-like changes is provided in Table 2.

**Table 2:** AD-driven scenarios of neuronal dysfunction and the corresponding model parameters changes

Scenario	Parameter	Parameter description	Parameter value			AD-mediated pathology
			Control condition	AD-like scenario	Contrast scenario	
**Pyramidal neuronal hyperactivity**						
**1A**	Vd_1_	Threshold potential of excitatory populations	7	6	8	A lower Vd_1_ value causes the excitatory populations to become hyperexcitable
**1B**	he(t) function (a1 and b1)	Excitatory post-synaptic potential (EPSP)	a1: 55 b1: 605	a1: 48 b1: 540	a1: 62 b1: 670	Increasing parameters *a* and *b* of the *h*_e_(*t*) function will increase the postsynaptic excitatory amplitude and duration of both the excitatory and inhibitory populations
**1C**	S	Global coupling factor	1.5	2.0	1.0	A higher global coupling factor results in stronger excitatory output (*E*(*t*)) multiplication and thus increased excitatory innervation of the excitatory population in the coupled neural masses
**Inhibitory neuronal dysfunction**						
**2A**	Vd_2_	Threshold potential of inhibitory populations	7	8	6	A higher Vd_2_ value causes the inhibitory populations to become hypoexcitable
**2B**	h_i_(t) function (a2 and b2)	Inhibitory post-synaptic potential (IPSP)	a2: 27.5 b2: 55	a2: 40 b2: 70	a2: 17.5 b2: 35	Higher values of parameters *a* and *b* of the *h*_i_(*t*) function will decrease the postsynaptic inhibitory amplitude and duration in the excitatory populations
**2C**	C2	Coupling from inhibitory to excitatory populations	3	2	4	A lower C2 value will decrease the inhibitory to excitatory coupling

Parameters Vd_1_ and Vd_2_ were part of a non-linear function that related the membrane potential to corresponding pulse densities. These parameters defined the firing threshold potential and thus were candidate model parameters to integrate AD-mediated increased excitability of the excitatory and decreased excitability of the inhibitory neuronal populations, respectively. The inhibitory and excitatory postsynaptic potentials (IPSP and EPSP) were modelled in the impulse response functions *h*_e_(*t*) and *h*_i_(*t*) and were suitable candidates to simulate AD-mediated enhanced excitatory neurotransmission (by disrupted glutamate homeostasis) or a reduced inhibitory neurotransmission (by a lower number of inhibitory postsynaptic receptors), respectively. The local inhibitory coupling coefficient (C2) determined the interaction between inhibitory and excitatory neuronal populations within a neural mass and was a good candidate to generate a model with AD-mediated reduction in inhibitory synaptic coupling strength and/or number of functional synaptic contacts to pyramidal neurons. The global coupling factor S multiplied the excitatory output (i.e., spike density) of one neural mass before it reached another neural mass (if coupled) and allowed simulation of an AD-mediated long-range increase of excitatory activity to other coupled excitatory neuronal populations.

##### Scenario 1: pyramidal neuronal hyperactivity

- 1A: (Intrinsic) pyramidal neuronal hyperexcitability. Different pyramidal neuronal excitability levels were obtained by adjusting the excitatory neuron firing threshold parameter Vd_1_(*t*). In the “healthy” or control condition, the threshold value for the excitatory (Vd_1_) neuronal populations had a value of 7 and this was altered to the value 6, meaning that the threshold was lower and we thus simulated a network with AD-like pyramidal neuronal hyperexcitability. The pyramidal neuron threshold potential was set to 8 to generate a contrast scenario with pyramidal neuronal hypoexcitability.

To test the effect of AD-like increased extracellular glutamate levels and thus a scenario with higher excitatory neurotransmission, we studied the effect of two different model parameters (scenario 1B and 1C).

- 1B: Increased excitatory postsynaptic potential. To simulate the effect of increased extracellular glutamate levels, we changed the EPSP curve that was modeled as the impulse response function *h*_e_(*t*) with parameters *a*_1_ and *b*_1_ (S2 Fig). Although increased neurotransmitter concentration is physiologically translated to higher EPSP frequencies in the postsynapse and not higher amplitude or duration, in this model, we regarded increased EPSP amplitude as synchronous EPSPs and thus a summation of multiple EPSPs. In control condition, a_1_ had a value of 55 and b_1_ of 605 s^−1^ that were set to 48 and 540 s^−1^ for AD-like increased EPSP amplitude and duration respectively (Table 2). Important to note here is that when we changed the EPSP curve, this not only affected the pyramidal (excitatory) neuronal population but also the inhibitory neuronal population, because both excitatory and inhibitory populations received excitatory input in the model. Changing the EPSP curve thus influenced both excitatory and inhibitory activity. The excitatory impulse response function *h*_e_(*t*) parameters *a*_1_ and *b*_1_ received a value of 42 and 670 to simulate a contrast scenario with decreased EPSP amplitude/duration.
- 1C: Increased excitatory to excitatory coupling. As alternative scenario of increased excitatory signals in the circuit (due to glutamate reuptake block by AD pathology), we could modulate the global coupling factor (*S*) between coupled neural masses. We increased the *S* value from 1.5 in control to a value of 2.0 in the AD-like scenario, which led to a stronger multiplication of the excitatory output signal (that is spike density (*E*(*t*)) between the pyramidal neuronal populations of two coupled neural masses and thus more excitatory input to the excitatory populations only). In the non-AD-like contrast scenario, the *S* parameter was set to a value of 1.0 to simulate reduced excitatory input towards the neural masses.

##### Scenario 2: Inhibitory neuronal dysfunction

- 2A: (Intrinsic) inhibitory neuronal hypoexcitability. Similar adjustments were made as in scenario 1A but now for the firing threshold of the inhibitory neuronal populations (parameter Vd_2_), i.e., the control scenario had a Vd_2_ value of 7, the AD-like scenario of inhibitory hypoexcitability received a Vd_2_ value of 8 (and thus a higher firing threshold), and the contrast-scenario received a Vd_2_ value of 6, reflecting inhibitory hyperexcitability.
- 2B: Decreased inhibitory post-synaptic potential. To simulate AD-like decreased inhibitory transmission and a reduced number of postsynaptic inhibitory receptors in the pyramidal neuronal population, we changed the amplitude and duration of the IPSP of the pyramidal population only (because the inhibitory neurons did not receive inhibitory input in the current model). Parameters *a*_2_ and *b*_2_ of the impulse response function *h*_i_(*t*) determined the IPSP shape and had a value of 27.5 and 55 s^−1^ in control condition (S2 Fig) and were adjusted to 40 and 70 s^−1^ to simulate AD-like reduced IPSP for a_2_ and b_2_ respectively. The parameters for the IPSP received a value of 17.5 and 35 s^−1^ to simulate a contrast scenario with increased IPSP (S2 Fig).
- 2C: Decreased inhibitory synaptic coupling. To simulate a loss of functional inhibitory synapses and therefore reduced inhibitory synaptic coupling strength, we adjusted the local inhibitory synaptic coupling coefficient C2 that determined the inhibitory to excitatory coupling strength. In control conditions, the C2 was 3, and to simulate AD-like loss of inhibition this parameter received a value of 2. The C2 parameter was increased to 4 in the non-AD-like contrast scenario to mimic stronger inhibition of excitatory synapses.

We chose to study the effects of each parameter modification separately to understand its specific contribution. First, we explored the effects of introducing insults on single (uncoupled) neural mass activity (S1 Table, S3 Fig, S4 Fig). Second, we repeated this for each scenario in the network model. Integrating the lesions in a single neural mass showed different effects than in a network of neural masses, indicating the unpredictability and importance of investigating AD mechanisms in a network. We ran simulations of MEG-like activity 50 times (except for the spike density analysis, which was repeated 10 times for each scenario), adequately capturing the variability of the output (see Fig. 4). The computational brain network model was programmed in Java and implemented in the in-house developed program BrainWave (version 0.9.152.12.26), written by C.J. Stam (latest version available for download at http://home.kpn.nl/stam7883/brainwave.html).

### Human data

#### Subjects

MEG data from a total of 36 participants were obtained from the Amsterdam Dementia Cohort of Alzheimer center Amsterdam (Amsterdam UMC, location VUmc). All subjects underwent a standardized screening including assessment of medical history, informant-based history, physical and neurological examination, neuropsychological evaluation, MEG, laboratory tests, MRI scanning, and lumbar puncture (and/or PET imaging) to quantify the levels of amyloid and tau in cerebral spinal fluid (CSF). An interdisciplinary clinical committee established a diagnosis [91]. We included healthy elderly subjects without cognitive impairment and with negative amyloid biomarker status (by CSF or PET imaging), although with a diagnosis of subjective cognitive decline (SCD) according to the standard diagnostic criteria [1, 46], as well as age- and gender-matched patients diagnosed with mild cognitive impairment (MCI) with evidence of amyloid pathology. MCI patients received a (semi-)annual clinical follow-up for 3 years after the initial visit to the clinic and 67% of these patients converted to AD dementia after 3 years. All participants gave written informed consent for use of their clinical data in future scientific research and the Amsterdam Dementia Cohort received approval by the Institutional Ethics Review Board of the VUmc.

#### MEG data acquisition and analyses

In short, as part of the standardized screening protocol, subjects underwent a two times 5 min resting-state eyes-closed MEG recording in a supine position in a magnetically shielded room (VacuumSchmelze GmbH, Hanua, Germany) using a 306-channel whole-head system (Elekta Neurmag Oy, Helsinki, Finland). Patients were instructed to close their eyes and lay still, but stay awake. A sample frequency of 1250 Hz was used, with an online anti-aliasing (410 Hz) and high-pass filter (0.1 Hz). We applied an offline temporal extension of Signal Space Seperation filter (tSSS) implemented in the MaxFilter software (Elekta Neuromag Oy, version 2.2.10), with a sliding window and correlation limit of 10s and 0.9 respectively. Raw data were visually inspected to select bad channels that were manually discarded before estimation of the SSS coefficients. The number of excluded channels varied between 1 and 12 channels. The head position relative to the MEG sensors was recorded continuously using the signals from four head-localization coils. Using a 3D digitizer (Fastrak, Polhemus, Colchester, VT, USA), we digitized the head-localization coil positions as well as the outline of the participant’s scalp (∼ 500 points). This scalp surface was used for co-registration with a structural (MRI) template that produced the best fit.

#### MEG source reconstruction

Source reconstruction was performed by an atlas-based beamforming approach [39]. Sensor signals were projected to an anatomical framework by means of automated anatomical labeling (AAL [87]) such that source-reconstructed neuronal activity for 78 cortical regions-of-interest (ROIs [31]; and two hippocampi were obtained. A centroid-based approach according to Hillebrand et al. [39] was applied to obtain representative single time series for each ROI. The sphere that best fitted the scalp surface was used as a volume conductor model to compute the beamformer weights and an equivalent current dipole was used as source model. The orientation of the dipole was chosen to maximize the beamformer output [72]. First, the broadband (0.5–70 Hz) normalized beamformer weights for the selected voxel were computed [14] and subsequently the broadband (0.5–48 Hz) time series for this voxel, i.e., a virtual electrode, was reconstructed (see [39] for details). The source-reconstructed time series were converted to ASCII files and five artifact-free, downsampled epochs (4096 samples, 13.2 s each) of the first 5-min MEG recording for each subject were used for further analysis.

### Outcome measures

**Fig. 3:**
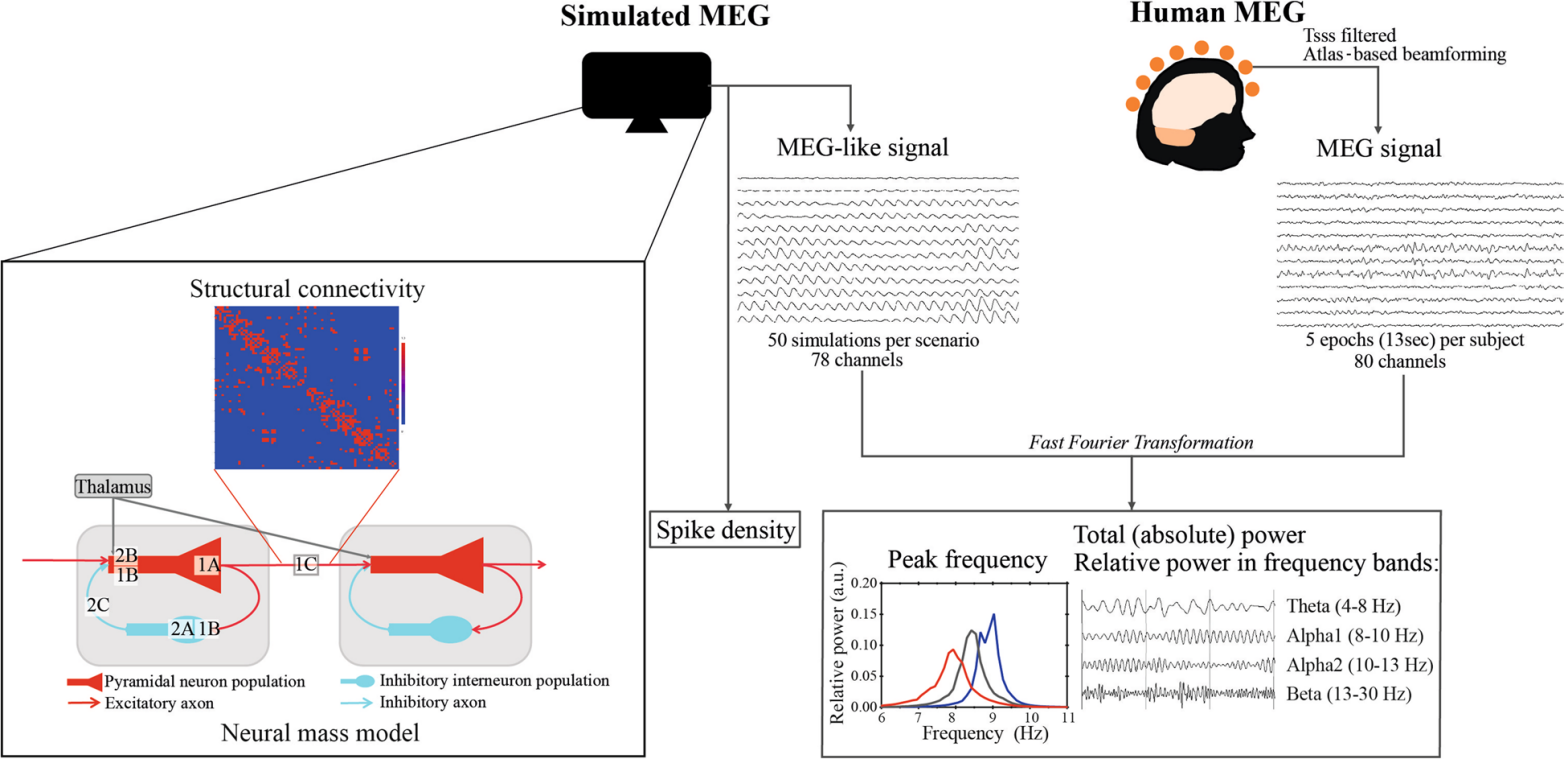
Simplified illustration of data generation and analyses of simulated MEG and human MEG. A computational brain model comprised of 78 neural masses coupled according to human DTI-derived binary structural connectivity matrix [31] was used to simulate whole-brain MEG-like oscillations. The simulated oscillations are derived from fluctuations in average membrane membrane potential of the excitatory neurons of each neural mass. We investigated the effect of different AD-driven neuronal function changes (see Fig. 2) on network oscillations and spike density of the pyramidal neuron populations. Fast Fourier Transformation was applied to simulated and human MEG data for spectral analysis. Human resting-state eyes-closed MEG was available for 18 prodromal AD patients (amyloid positive patients diagnosed with mild cognitive impairment) as well as 18 age- and gender-matched elderly control subjects

Similar spectral analyses have been applied to both simulated and human MEG data (Fig. 3) using BrainWave. Each virtual electrode or neural mass in the model network was subject to Fast Fourier Transformation to derive a power spectrum, the relative power in commonly used frequency bands delta (0.5–4 Hz), theta (4–8 Hz), lower alpha 1 (8–10 Hz), higher alpha 2 (10–13 Hz), beta (13–30 Hz) and gamma (30–45 Hz), total power (absolute broadband power, 0.5–48 Hz), and peak frequency (between 4 and 13 Hz). In this study, we focused on relative alpha 1 and alpha 2 power, with additional adjacent relative theta and beta power. Gamma and delta bands were excluded from the analysis because these bands are frequently contaminated by physiological artifacts in human data and because we were primarily interested in effects on the dominant alpha rhythm as empirical hallmark in neurophysiological AD data.

For human data, the outcome measures were averaged over 5 epochs per subject and 78 ROIs to obtain whole-brain MEG characteristics prior to group statistics. Peak frequency was calculated for parieto-occipital regions only. For simulated MEG data, we obtained ‘whole-brain’ output by averaging over all 78 coupled neural masses for each iteration and subsequently averaged the outcome measures over all iterations per scenario. To visualize changes in simulated spectral peak upon AD-like insults irrespective of amplitude, each average power spectrum was normalized to a total (absolute) power of 1. Also, the relative power was plotted over the dominant oscillatory frequency range only to make changes in the oscillatory peak visible. Spike density captured the neuronal activity of the pyramidal neuron population within the neural masses for each time point with a sampling time of 0.002 seconds and was reported as the average spike density over the whole brain (all 78 neural masses) for 10 iterations.

### Combining simulated and human data

This study first tested the hypothesis that AD-like neuronal mechanisms increase the excitatory neuronal activity and thus an increase in spike density compared to control condition and that contrast (non-AD-like) scenarios decrease the spike density. Second, a visual inspection of the average changes (while taking into account the variances) in modeled brain oscillatory power and peak frequency was performed. Third, based on previous (pre-)clinical findings, the hypothesis that human prodromal AD patients’ MEG have a characteristic global slowing of oscillations, at least involving higher relative power in slower (theta) frequency bands, was tested. In addition, total (absolute broadband) power was analyzed, a potential indicator of E/I imbalance and neuronal network hyperexcitability. Fourth, simulated brain activity was compared to human data. Although one could prefer to fit the model to empirical data, this is a computationally expensive method and introduces other problems such as how to achieve and determine a good fit. Moreover, in our opinion, aiming to reach an optimal fit between modeled and empirical data can be considered over-interpretation of the data and goes beyond the purpose of this particular study, i.e., we regarded the demonstration of a more general(izable) link between neuronal hyperexcitation and oscillatory slowing to be more convincing than a perfect fit in this specific dataset, also given the natural variability of neurophysiological data. Therefore, model and human MEG data was compared in a qualitative way on key parameters that have also been established in neurophysiological AD literature in the past decades [33, 34, 69]. Finally, the findings were summarized into three subcategories: oscillatory behavior (that can be slower/faster/not altered in the diseased state compared to control condition), neuronal activity (indicating whether the spike density levels of the excitatory neuronal populations in the model were higher/lower/not different from control condition), and total power (reflecting higher/lower/similar absolute broadband power as control condition). Because the model generatds mainly alpha activity we had to be careful in making conclusions about and to not over-interpret individual band-pass power changes upon AD-like neuronal dysfunction. Therefore, we visually inspected the data and provided this composite oscillatory behavior outcome measure that may indicate global oscillatory slowing even in the presence of some increase in the faster beta power.

### Statistical analyses

Statistical software package SPSS version 25.0 for Mac was used for statistical analyses of human MEG data. Subject characteristics were compared between groups with independent samples *t*-tests. Spectral measures were visually checked for normal distribution using histograms and were subsequently compared with independent samples *t*-tests (not assuming equal variance). Statistical significance was determined at *p* < 0.05 for whole-brain analyses. Simulated MEG data were not statistically tested because of the following reasons: (1) as many simulations as needed can be computed to obtain good statistical power and (2) model parameter values could be relatively arbitrarily chosen and therefore concluding whether a small change in outcome was more or less relevant than a large effect can be considered over interpretation. However, error bars and all individual (average) data points were included in the figures.

## Results

### Pyramidal neuronal hyperactivity

#### Scenario 1A: (Intrinsic) pyramidal neuronal hyperexcitability

**Fig. 4:**
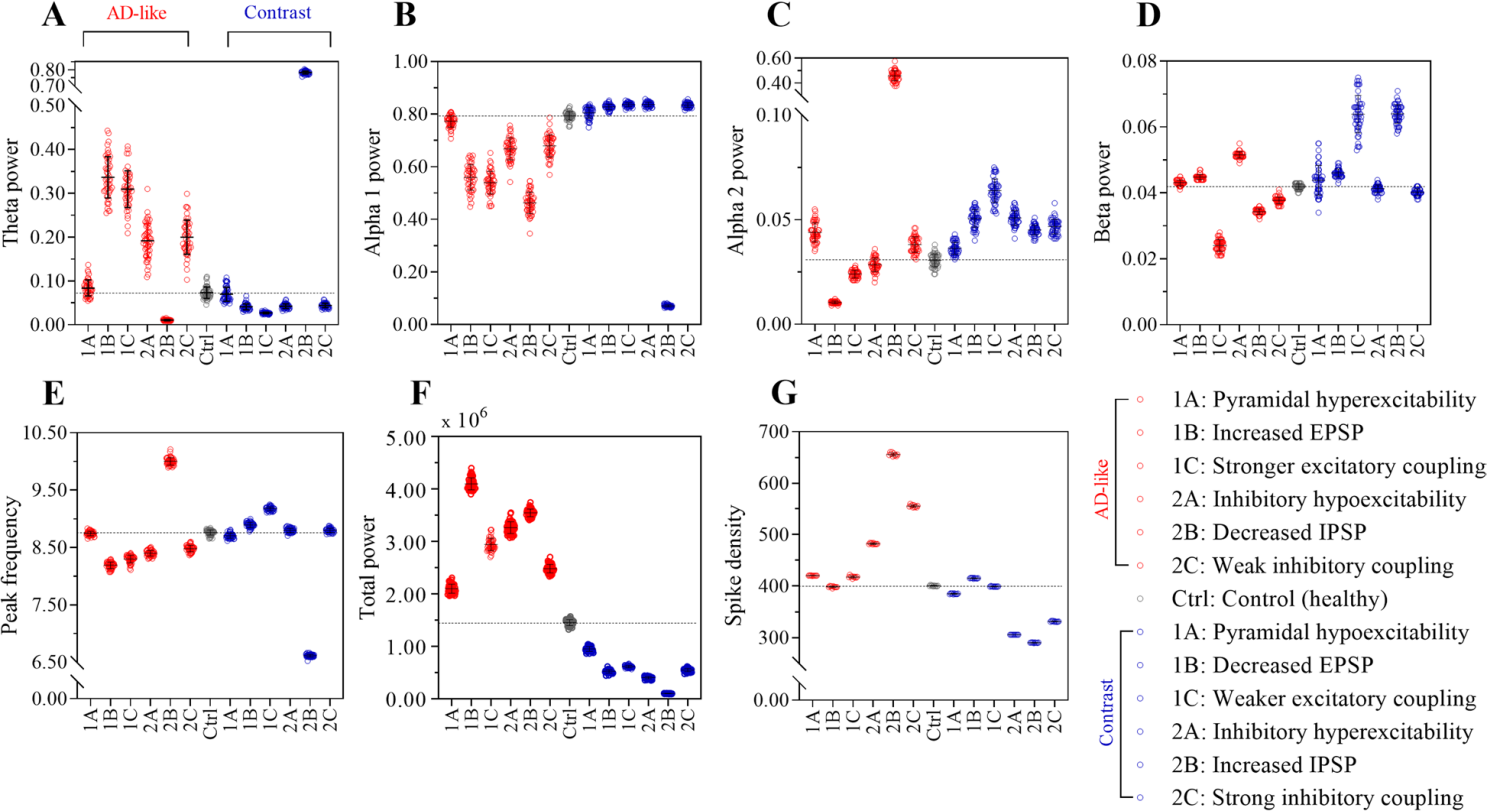
Oscillatory behavior after AD-mediated neuronal dysfunctions. This figure shows the effect of simulated AD-like microscale mechanisms (in red) on large-scale outcome measures. The results of the healthy control scenario  are plotted in grey and the opposite, non-AD-like, mechanisms (contrast scenarios) are shown in blue. For each AD-like or contrast scenario, we modified a single model parameter (see Table 2) and analyzed the simulated MEG. **A**–**D** Relative power in four frequency bands of interest (theta (4–8 Hz), alpha 1 (8–10 Hz), alpha 2 (10–13 Hz), beta (13–30 Hz)). **E** Peak frequency shows the dominant frequency between 4 and 13 Hz. **F** Total (absolute broadband (0.5–48 Hz)) power. **G** Spike density indicates the spiking activity of the pyramidal neuronal populations in the network over a certain time period. Each dot represents the average (whole-brain) value over all 78 ROIs for 1 iteration. Number of iterations: 50, except for spike density that has been iterated 10 times. Black lines are mean and standard deviation (SD) values over all iterations per scenario. *X*-axis represent the different scenarios (see legend). Note the differences in *y*-axis for the distinct relative frequency bands; i.e., alpha 2 and beta frequencies have more than 10 times lower power values than relative alpha 1 power

Increased pyramidal neuronal excitability (i.e., hyperexcitability) resulted in higher relative power in the theta, (Fig. 4A), alpha 2 and beta bands (Fig. 4C and D), as well as lower relative power in the alpha 1 band and a lower peak frequency (Fig. 4B and E) compared to the control condition. Furthermore, AD-like pyramidal neuronal hyperexcitability was associated with higher total power and spike density (Fig. 4F and G) compared to control.

In contrast, decreased excitability of the pyramidal neurons (i.e., hypoexcitability) resulted in higher average relative power of alpha 1 (Fig. 4B), alpha 2 and beta bands (Fig. 4C and D) compared to control, but did show a difference in relative theta power compared to control (Fig. 4A). Pyramidal neuronal hypoexcitability also resulted in a slower peak frequency (Fig. 4E) but this was associated with lower total power and reduced spike density (Fig. 4F and G) compared to control.

#### Scenario 1B: Increased excitatory postsynaptic potential

Prolonged EPSP caused higher relative power in the theta and beta frequency bands (Fig. 4A and D) and a decrease in alpha 1 and 2 band (Fig. 4B and C) compared to control. Furthermore, increased EPSP resulted in a lower peak frequency and higher total power (Fig. 4E and F) but a small decrease in pyramidal neuron spike density (Fig. 4G) compared to control.

In contrast, decreased EPSP amplitude and duration resulted in lower relative power in theta frequency band (Fig. 4A) and higher relative alpha 1, alpha 2 and beta power (Fig. 4B and C and D) compared to control. In addition, decreased EPSP amplitude and duration showed a higher peak frequency (Fig. 4E) and spike density of the pyramidal neuron population (Fig. 4G) but lower total power (Fig. 4F) compared to control.

#### Scenario 1C: Increased excitatory to excitatory coupling

An increased excitatory to excitatory coupling between the neural masses resulted in higher relative theta and beta power (Fig. 4A and D) and lower relative alpha 1 and alpha 2 power (Fig. 4B and C) compared to control. The peak frequency was also lower (Fig. 4E) and the total power and spike density were increased (Fig. 4F and G) compared to control.

In contrast, lower excitatory global coupling resulted in lower relative theta and beta power (Fig. 4A and D) and increased relative alpha 1 and alpha 2 power (Fig. 4B and C) compared to control. The peak frequency was higher (Fig. 4E) and total power and spike density were decreased (Fig. 4F and G) compared to control.

### Inhibitory neuronal dysfunction

#### Scenario 2A: Decreased inhibitory interneuron excitability

A decrease in excitability of the inhibitory interneurons (i.e., hypoexcitability) resulted in higher relative theta (Fig. 4A) and beta power (Fig. 4D) and lower power in relative alpha 1 (Fig. 4B) and alpha 2 (Fig. 4C) frequencies compared to the control condition. Inhibitory hypoexcitability furthermore resulted in lower peak frequency and higher total power (Fig. 4E and F) and spike density of the pyramidal population (Fig. 4G) compared to control condition.

Increased excitability of inhibitory interneurons resulted in lower relative theta and beta power (Fig. 4A and D) and higher relative alpha 1 (Fig. 4B) and alpha 2 (Fig. 4C) power in comparison with control condition. Furthermore, the data presented a higher peak frequency (Fig. 4E) and decrease in total power (Fig. 4F) and pyramidal neuronal spike density (Fig. 4G) upon decreased inhibitory neuronal excitability.

#### Scenario 2B: Decreased inhibitory postsynaptic potential

Decreased IPSP amplitude and duration caused lower relative theta, alpha 1, and beta power (Fig. 4A, B, and D) and increased alpha 2 power (Fig. 4C) compared to control scenario. Furthermore, higher peak frequency and higher total power (Fig. 4E and F) and spike density (Fig. 4G) resulted from decreased IPSP in comparison with the control condition.

Increased amplitude and duration of the IPSP resulted in higher relative theta, alpha 2, and beta power (Fig. 4A, C, and D) and lower relative alpha 1 power (Fig. 4B) compared to the control condition. Also, increased IPSP showed a lower peak frequency (Fig. 4E) as well as lower total power and pyramidal neuron spike density (Fig. 4F and G) compared to the control scenario.

#### Scenario 2C: Decreased inhibitory synaptic coupling

Reduced inhibitory to excitatory coupling resulted in greater power in relative theta and alpha 2 frequency bands (Fig. 4A and C) and lower relative alpha 1 and beta power (Fig. 4B and D) in comparison with the control scenario. Subsequently, the simulated EEG signals for this scenario showed lower peak frequency (Fig. 4E) and higher total power (Fig. 4F) and spike density (Fig. 4G).

In contrast, increasing inhibitory to excitatory coupling caused lower relative theta and beta power (Fig. 4A and D) and higher alpha 1 and alpha 2 power (Fig. 4B and C). The peak frequency was also higher compared to control condition (Fig. 4E) and a lower spike density could be observed (Fig. 4G).

### Simulated MEG power spectra

**Fig. 5:**
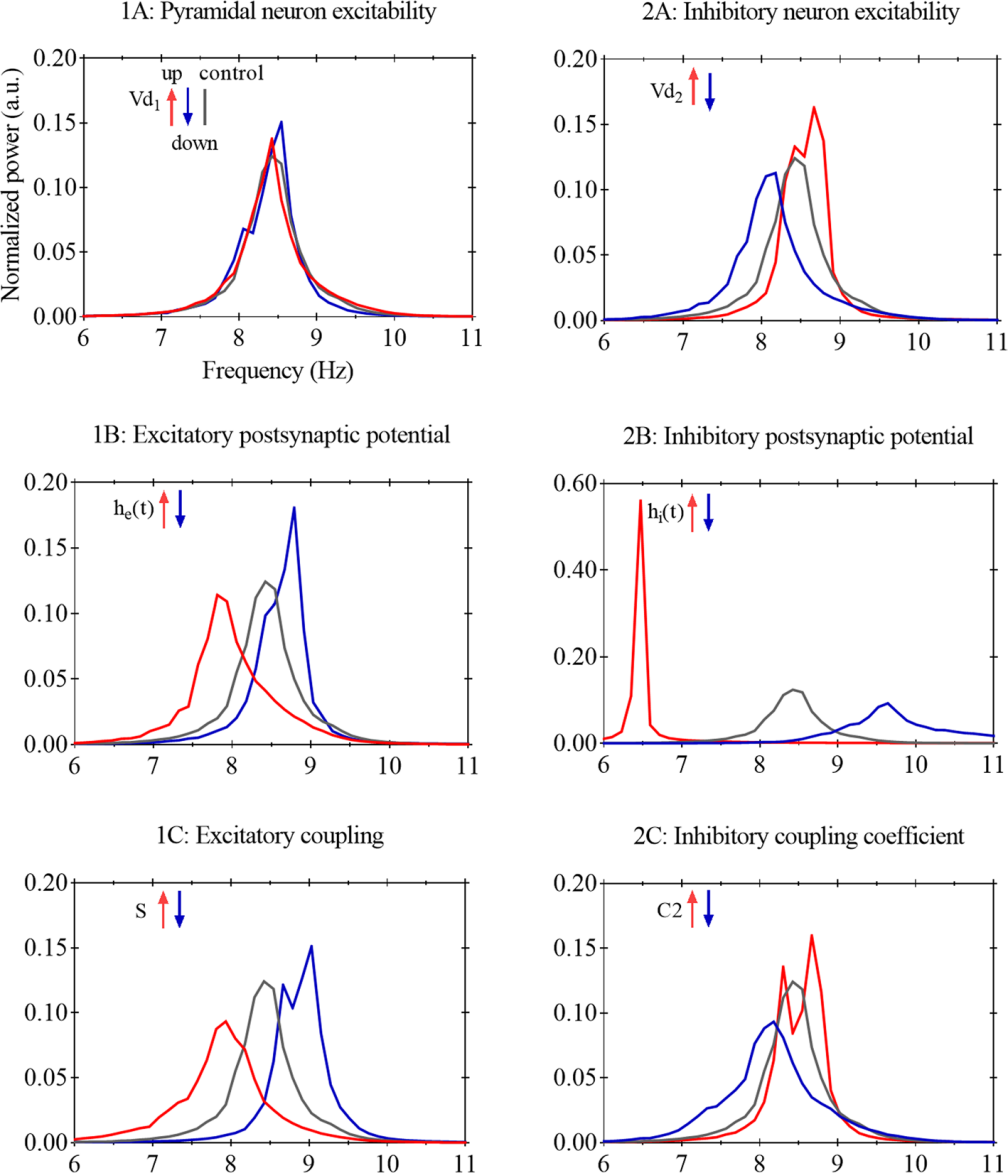
Normalized average power spectra of simulated MEG across AD-like, healthy control and contrast scenarios. The average power spectra were normalized to total power and averaged over 50 iterations. For each AD-like or contrast scenario, we modified a single model parameter (see Table 2), by increasing (up: shown in red) or decreasing (down: shown in blue) this parameter. Of note, interpretation of the direction (up/down) of parameter modification is intuitively, such that ‘up’ means stronger coupling or hyperexcitability (which is actually derived by lowering the threshold). Control (or healthy) condition is shown in grey. Note that a different *y*-axis is used for scenario 2B

Figure 5 shows the normalized average power spectra for all scenarios. From this figure, one can appreciate that multiple scenarios had higher power in more slow frequencies (i.e., oscillatory slowing) whereas other scenarios showed the opposite pattern: an increase in power in the faster frequencies across the alpha 1/2 frequency range.

### Human MEG

Table 3 shows the main characteristics of both subject groups. Comparing subject characteristics between 18 amyloid-negative SCD patients (SCD−) and 18 age and gender-matched amyloid-positive MCI subjects (MCI+) (Table 3) shows that the MCI+ patients had on average significantly lower mini-mental state examination (MMSE) scores (*p* < 0.01) than SCD− subjects (Table 3).

**Table 3:** Subject characteristics

	SCD−	MCI+
**N**	18	18
**Female/male**	10/8	9/9
**Age (y)**	64.2 (± 6.1)	64.1 (± 6.2)
**MMSE score**	27.8 (± 2.1)	25.8 (± 1.9)**

Values are mean and standard deviation, unless stated otherwise. Differences were tested with independent samples *t*-tests

*SCD* subjective cognitive decline, *MCI* amnestic mild cognitive impairment with positive amyloid biomarkers for Alzheimer’s disease, *M/F* male/female, *MMSE* Mini-Mental State Examination

^**^p < 0.01

**Fig. 6:**
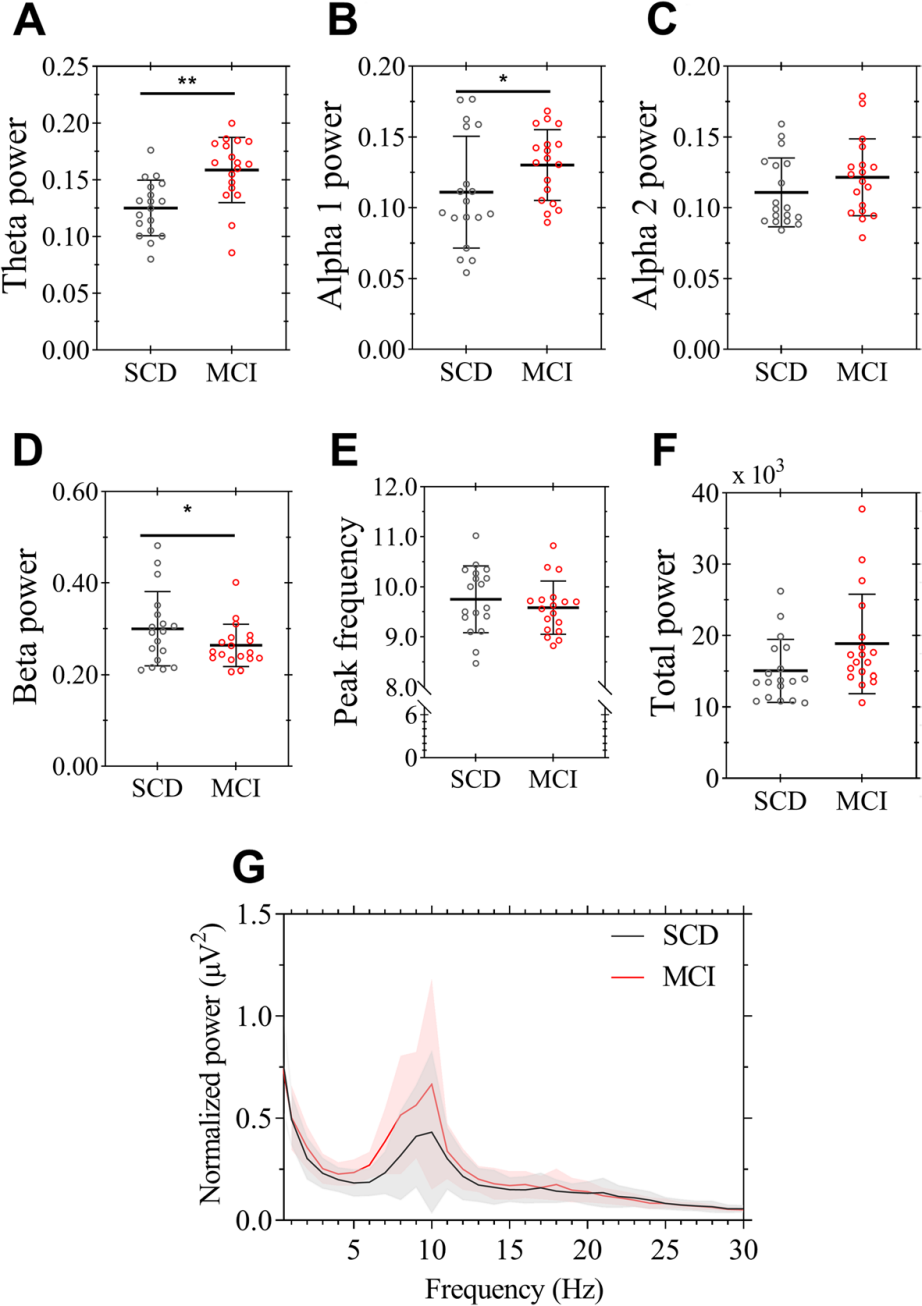
Human MEG spectral analyses. Shown are median and IQR of the relative power in commonly used frequency bands (**A** theta (4–8 Hz), **B** lower alpha 1 (8–10 Hz), **C** higher alpha 2 (10–13 Hz), **D** beta (13–30 Hz)) as well as the **E** peak frequency (that represents the posterior dominant frequency within the 6–13 Hz band), and **F** total power (absolute broadband (0.5–48 Hz)) power for 18 MCI+ patients (shown in red) and 18 elderly controls (SCD−, shown in grey). Each dot represents the average value for each subject over 5 epochs and 80 channels. **G** The power spectrum is normalized such that the total power is 1 for each group. Shown are the mean and standard deviation per group. **p < 0.01, *p < 0.05

Whole-brain MEG spectral measures are compared between MCI+ and SCD− subjects (Fig. 6). Independent samples *t*-tests for the global MEG measures showed that relative theta (*t*(34) = 3.95, *p* < 0.01) and relative alpha 1 power (*t*(34) = 2.13, *p* < 0.05) was significantly higher in the MCI+ group (Fig. 6A and B), whereas the beta power was significantly lower (*t*(34) = −2.04, *p* < 0.05) compared to SCD− subjects (Fig. 6D). The groups did not differ in peak frequency or relative alpha 2 power (Fig. 6E and C). Total (absolute broadband) power was higher in MCI+ subjects compared to SCD− subjects, but did not reach significance (*t*(34) = −1.958, *p* = 0.060) (Fig. 6F). The normalized power spectra (Fig. 6G) indicate relatively higher power across the theta and lower alpha frequencies in the MCI group compared to the control group, albeit with a large variability (not statistically tested).

### Combining simulated and human MEG data

All of the simulated AD-like scenarios show a higher average spike density of the pyramidal neurons (that cannot be measured directly in humans using MEG), except for scenario 1B (increased EPSPs). Oscillatory slowing, i.e*.*, a shift in relative power from higher frequencies to lower frequencies, mainly theta power, was observed in MEG of MCI patients (Table 4) as well as in the majority of the empirically informed AD-like scenarios (except for scenario 2B: decreased IPSP) compared to controls. Of the five AD-like scenarios with higher spike density, four also had slower oscillatory activity similar to human AD patients. Human MCI patients’ MEG data showed a trend towards higher total (absolute broadband) power, which was consistently found in all simulated brain activity of the AD-like scenarios. Across the contrast (non-AD-like) scenarios, one scenario showed higher spike density compared to the control condition but this was not linked to oscillatory slowing or higher total power. One contrast scenario showed slower oscillatory behavior, but this was not complemented with higher spike density or higher total power.

**Table 4:** Summary table of the main outcome measures for human MEG and simulated MEG

	Human AD	AD-like scenarios						Contrast scenarios					
		1A	1B	1C	2A	2B	2C	1A	1B	1C	2A	2B	2C
Parameter		Vd1	EPSP	S	Vd2	IPSP	C2	Vd1	EPSP	S	Vd2	IPSP	C2
Direction		up	up	up	down	down	down	down	down	down	up	up	up
Neuronal activity	n.a.	Higher	Lower	Higher	Higher	Higher	Higher	Lower	Higher	Lower	Lower	Lower	Lower
Oscillatory behavior	Slower	Slower	Slower	Slower	Slower	Faster	Slower	Faster	Faster	Faster	Faster	Slower	Faster
Total power	Higher (n.s.)	Higher	Higher	Higher	Higher	Higher	Higher	Lower	Lower	Lower	Lower	Lower	Lower

This table shows the direction of change in MEG outcome measures for human MCI and simulated early AD-like or its opposite (contrast) scenarios. Neuronal activity (that is spike density of pyramidal neurons) can only be assessed in model data and show an increase in AD-like scenarios mainly. Of note, the interpretation of the direction (up/down) of parameter modification is intuitive, such that “up” of means hyperexcitability (which is actually derived by lowering the threshold) or stronger coupling. N.a. not available, N.s. not significant, AD Alzheimer’s disease

## Discussion

Previous in vitro and in vivo studies in experimental models of AD revealed early increases in neuronal excitability and cellular firing rates associated to AD pathology [9, 10, 38, 53, 58, 99], but direct evidence for these findings in human AD patients is limited and difficult to acquire [49]. This study used a computational dynamic brain network model to introduce different AD-dependent mechanisms of neuronal hyperactivity on a cellular level, investigated its effect on a physiological network level, and compared this with large-scale human MEG data of early-stage AD patients. Of the hypothesized mechanisms of AD, the majority indeed showed neuronal hyperactivity complemented with slowing of the oscillations (i.e., an increase in slower frequencies, theta power mainly, and decrease in faster frequencies) and thus replicated human AD patients’ MEG. These scenarios included hyperexcitable excitatory neurons, hypoexcitable inhibitory interneurons, stronger long-range excitatory coupling, and weaker local inhibitory coupling. In contrast, non-AD-like scenarios showed opposite effects.

### AD-driven neuronal hyperactivity explains oscillatory behavior in early disease stages

The modeled AD-like scenarios were based on important candidate mechanisms from recent AD literature to increase their plausibility. Previous reports provided evidence for altered excitability of pyramidal neurons in early stages of AD [11, 29, 99]. Indeed, the data showed that pyramidal neuronal hyperexcitability (scenario 1A) result in higher pyramidal spike density and lead on a network level to behavior that resemble aspects of the electrophysiological abnormalities of prodromal AD patients, including higher total broadband power, higher relative theta power, lower relative alpha 1 power, and a lower peak frequency (i.e., a slowing of the dominant brain rhythm). Contradictory to a slower oscillatory profile with reduced alpha 1 power in this and other AD-like scenarios, the human prodromal AD patients showed an increase in alpha 1 power. However, we have concluded that this group has slower oscillations because it shows increased theta and reduced beta. Also, in humans a shift to alpha 1 power may be a part of the oscillatory slowing process because the peak generally lays in the higher alpha 2 band. This is in contrast to the “healthy” alpha peak in the alpha 1 band in simulated data, and thus a slowing will cause a reduction of the alpha 1 instead.

Although one computational model study linked hyperexcitable pyramidal neurons to oscillatory slowing previously [80], it is not a trivial finding, because various other scenarios resulted in very different, incompatible oscillatory behaviors. The finding that neuronal hyperactivity in AD is likely explained by hyperexcitable pyramidal neurons is in line with results from human induced pluripotent stem cell (iPSC)-derived neurons of AD mutation carriers, showing enhanced excitability compared to wild-type neurons [29]. When looking at the neurophysiological epilepsy literature, where neuronal hyperexcitability in seizures is often accompanied by pronounced theta activity (e.g., the “Risinger” rhythm in temporal epilepsy) [84], this may reflect a similar mechanism, albeit with a different cause and disease course. Intriguingly, a low dose of the anti-epileptic drug levetiracetam has shown promise to reduce hyperactivity and improve cognitive performance in predementia AD patients [4, 5] and several clinical trials testing levetiracetam treatment to reduce neuronal hyperexcitabilty in different populations of AD patients are ongoing (summarized in [85]). Further research is required to increase our understanding of anti-epileptic treatment effects in human AD patients.

Neuronal hyperexcitation through disturbed glutamatergic homeostasis in AD has gained increasing attention in the literature [37, 99]. This was simulated in two ways: (1) by increasing the amplitude and duration of the post-synaptic excitatory impulse response (scenario 1B) and (2) by increasing the global excitatory coupling strength between coupled neural masses (scenario 1C). Although scenario 1B generated a slowing of the oscillations, similar to our prodromal AD patients, this was associated with a (slightly) lower spike density, rather than neuronal hyperactivity. This may be found because not only pyramidal neurons but also inhibitory interneurons received increased EPSPs, which have tipped the E/I balance to an inhibition dominated network and reduced neuronal activity. The slowing of oscillations is likely a result of an increase in the length of the postsynaptic currents. Interestingly, when testing a stronger increase in EPSPs, spike density didincrease and led to a global slowing although with a very strong increase in total power, suggesting that a scenario of strongly increased EPSP is not very likely a phenomena of early stage AD (that only showed a trend towards higher total power). In contrast, stronger excitatory coupling (scenario 1C) resulted in neuronal hyperactivity, oscillatory slowing, and higher total power which provides support for an AD-mediated neuronal hyperexcitation and network dysfunction through disturbed glutamate homeostasis. Overall, the observed likelihood of hyperexcitability and hyperexcitation being involved in oscillatory slowing, as well as the seemingly beneficial effect of reducing hyperexcitability point from different angles towards its role in AD pathophysiology.

### Disinhibition leads to neuronal hyperactivity and AD-like oscillatory slowing

Inhibitory interneurons are known for their critical role in synchronizing neuronal activity and the generation of neuronal oscillations [42, 86]. Because their dysfunction is increasingly associated with AD [2], we also explored network-effects of AD-driven disinhibition. In a network of neural masses, all three disinhibition scenarios caused an increase in neuronal activity. Furthermore, inhibitory interneuron hypoexcitability (scenario 2A) and reduced inhibitory to excitatory coupling (scenario 2C), but not reduced IPSP (scenario 2B), resulted in oscillatory slowing. Other computational modeling studies also linked local disinhibition-mediated neuronal hyperactivity to increased theta oscillatory activity in the network [16, 80]*.* This study explored multiple mechanisms of inhibitory dysfunction based on empirical studies (as well as scenarios with opposite parameter changes) and used a model that generated realistic brain oscillations that allowed for a qualitative comparison to human data. Of interest, a reduction in the IPSP (of pyramidal neurons, scenario 2B) resulted in neuronal hyperactivity, but not oscillatory slowing. This finding indicates that the postsynaptic activity, possibly due to an altered number of inhibitory receptors of the pyramidal neurons in AD, is most likely not causing early-stage oscillatory slowing. In cultured human neurons, patch-clamp recordings revealed a significant decrease in the frequency of IPSPs in AD neurons, but not a change in amplitude [29], as was also reported in another study using a transgenic AD animal model [92]. Perhaps, reducing the duration and thus the total current of the IPSP to simulate reduced inhibition is not the optimal model parameter change to replicate the empirical studies, but rather a reduction in frequency of inhibitory impulses is more accurate. Taken together, these findings confirm previous experimental results and propose inhibitory dysfunction, in particular inhibitory interneuron hypoexcitability and decreased inhibitory synaptic coupling strength, as important contributor of neuronal hyperactivity and slowing of brain activity in early stages of AD.

### Oscillatory slowing due to suppressed neuronal activity in AD

Although scenarios of neuronal hyperactivity were most frequently linked to oscillatory slowing on the larger-scale, two scenarios showed both lower spike densities as well as oscillatory slowing, including the AD-like increased EPSP scenario (1B) and the contrast, non-AD-like, increased IPSP scenario (2B). Whereas the disturbed glutamate reuptake hypothesis was previously proposed as early-stage disease mechanism [29] and the increased EPSP model scenario replicated part of the human early stage AD patient findings, this scenario may be a more plausible explanation for later stages of AD. Advanced AD is characterized by more extensive spectral slowing that is intuitively and more commonly associated with neuronal hypoactivity [8]. Reduced neuronal activity is more likely a phenomenon of neurodegeneration and linked to tau pathology in more severely affected AD patients. Not only transgenic tau mice showed suppressed neuronal activity measured with in vivo calcium imaging in the parietal cortex [8], but clinical observations from fMRI and fluordeoxyglucose-PET in human AD patients in more severe disease stages also showed cortical and hippocampal hypoactivity and hypometabolism [22, 76]. Moreover, de Haan et al*.* detected neuronal hypoactivity and oscillatory slowing in the end stage of the activity dependent degeneration model [16]. Possibly, prolonged EPSP and increased IPSP represent mechanisms of neuronal hypoactivity and more severe oscillatory slowing in later disease stages.

### Neuronal hypoactivity in a network cannot explain MEG signals in early AD

Furthermore, contrast (non-AD-like) scenarios were explored and expected to most likely not explain human MEG data. Indeed, that is what the data presented here suggest, illustrating that oscillatory slowing is not a trivial outcome of merely changing some model parameter settings but this is more a specific large-scale outcome of AD-mediated neuronal dysfunction. One a-priori defined non-AD-like mechanism is of interest in particular and involved the hyperexcitable inhibitory neurons (2A). Although contradictory to the many other AD animal studies, Hijazi et al. [38] discovered inhibitory interneuron hyperexcitability (in PV+ cells specifically) in a transgenic AD animal model prior to hyperactivity of the pyramidal neurons [38]. Possibly, this scenario manifests in AD patients in an even earlier stage, without any or only subjective cognitive complaints. Based on our findings, this is translated on a network level into higher relative power in the faster frequencies, alpha 2 in particular, as well as a reduction in total power. Although investigated in a small sample, EEG analyses of presymptomatic *PS1* mutation carriers have shown higher power in the relative alpha 2 band and lower power in the theta band [23, 56, 57] compared to healthy subjects, potentially indicative of preclinical hyperexcitable inhibitory neurons. Alternatively, the lack of different interneuron subtypes in the model and a distinction between feedback or feedforward inhibition loops may have influenced the results.

### Oscillatory slowing due to neuronal hyperactivity requires network connectivity

Importantly, the effect of introducing AD-like and its opposite microscale pathophysiology in uncoupled, single, neural masses is different from that in a neural mass network (S1 Table, S3 Fig, S4 Fig). Similar to the network model, AD-like scenarios in a single, uncoupled, neural mass cause neuronal hyperactivity as well as higher total power mainly. However, this hyperactivity is frequently linked to higher power in the alpha 2 band and resulted in a higher peak frequency as well, and thus not replicated oscillatory slowing. The discrepancy in results indicate that microscale pathology may have different impact with increasing scale and complexity, which strengthens our choice to integrate single neurobiological substrates within a network model. Multiscale network modeling is challenging, but will ultimately increase our understanding of global network disruption and cognitive deterioration, not only in AD but also in other disorders. Future studies could aim to assess the influence of network topology by comparing scenarios running on different network types.

### Total power as potential biomarker of hyperactivity

Model data show a consistent increase in total power in all AD-like scenarios and, therefore, it may yield promise as a large-scale biomarker of early-stage AD. A clear trend towards higher total power levels in MCI patients can be appreciated from the human data. However, we cannot conclude that higher total power is merely a consequence of increased spiking of the pyramidal neuron population based on our findings. Total power in MEG is less sensitive to inter-individual differences in environmental settings than EEG but could still be influenced by non-physiological differences between groups. Future studies of total power as well as novel algorithms that could infer E/I balance from large-scale electromagnetic physiological signals (such as the functional E/I measure [6]) in early affected brain regions may ultimately provide direct support for the early stage neuronal hyperactivity in AD hypothesis and help to find relevant entry points for therapeutic targets [6, 27, 94].

### Strengths and limitations

According to the motto “a model should not be too simple, nor too complex”, one of the strengths of this study is that it employed a computational model that generates realistic macroscale neurophysiology, allowing for qualitative comparison to human data, yet is sufficiently straightforward to systematically explore meaningful parameter changes. The proposed AD-like mechanisms were based on empirical studies and are introduced in a network of neural masses, allowing to translate findings from multiple scales and models and testing opposite scenarios to make the AD-like mechanisms more probable. Human MEG recordings were analyzed in source space and involved data of well-characterized and matched subjects, although ideally we would have compared the MCI to cognitively elderly healthy control subjects without a diagnosis of subjective cognitive decline. This study is also constrained by some methodological choices. Analyzing narrow frequency bands of model data is not as informative as for human data, but here the primary interest of this study is to find mechanisms that underlie general, well-established AD hallmarks such as slowing of the posterior dominant alpha rhythm. This phenomenon could be reliably reproduced and interpreted by the model, including a reduction in peak frequency that was expected based on previous AD literature (reviewed in [24]). These very robust neurophysiological parameters have guided the model choice for this particular study. Another limitation is that only known pathophysiological effects of amyloid-β were introduced, because microscale studies of neuronal dysfunction have mainly been performed in amyloidosis models of AD and amyloid-β is (assumed) one of the earliest measurable indicators of AD (Jack [45]). Other factors such as inflammation and hyperphosphorylated tau may also influence the activity of neurons [44, 99] and their contribution to network abnormalities should also be explored. For simplicity, this study tested single parameter changes to find different contributors of network dysfunction in AD, although multiple effects of the toxic oligomers can occur simultaneously and likely influence each other. Another limitation is that regional vulnerability in AD is not considered, regardless of the well-known specific spreading patterns of amyloid-β and vulnerability of network hubs in AD [7, 35]. However, interpreting the effect of microscale changes on a global network level is already challenging, and considering spatial differences as well would introduce another layer of complexity.

### Future directions

Because this study attempted to relate scales that are challenging to combine in experimental practice, various follow-up endeavors can be imagined. First, replicating the current findings in more sophisticated models that replicate an extended number of empirical MEG phenomena (such as neuronal plasticity and region specific oscillatory frequencies) may extend our knowledge in regard of regional effects across multiple frequency bands and robustness of the network dynamics to AD-like perturbation on smaller scales [81]. Second, applying higher-order measures to model data, such as analysis of functional connectivity and network organization, could increase the likelihood of contribution of the currently proposed mechanisms to network imbalance in early stage AD, in particular because functional connectivity may be a biomarker of underlying neuronal hyperexcitability, considering that MCI is increasingly associated with hyperconnectivity ([24, 55]). Third, although a complication to the model, one can incorporate AD-like damage to the network over time to study directionality of effects as has been reported earlier [16], along with potential counter-mechanisms [18], which were not yet based on experimentally observed pathophysiological mechanisms [32]. Fourth, studying neuronal network activity of human iPSC-derived AD neurons in vitro as well as in vivo mesoscale data from animal models of AD would provide the missing link to bridge small-scale phenomenon to macroscale network changes in human patients.

## Conclusions

This study linked current empirical data from different scales and showed that molecular and cellular findings from AD animal models very likely explain network abnormalities in human prodromal AD patients and are thus mechanisms of therapeutic interest. Additional studies are required to find robust large-scale biomarkers of cellular hyperexcitability and E/I imbalance in early AD.

## Supplementary Information

**Additional file 1: **The neural mass model. Single neural mass analyses. Parameter range. **Table S1.** Single neural mass model. **Fig. S1.** Example traces of simulated MEG signals in control condition and several AD-like (and their contrast) scenarios. **Fig. S2.** The excitatory and inhibitory post-synaptic potentials (EPSP and IPSP). **Fig. S3.** Neuronal activity and total power in AD-like and contrast scenarios in a single, uncoupled, neural mass model. **Fig. S4.** Simulated power spectra for AD-like and contrast scenarios in a single, not-coupled, neural mass model. **Fig. S5.** Robust changes in outcome measures over a range of values for each relevant model parameter.

## Data Availability

The datasets generated by the computational brain model during the current study are available in the Zenodo repository, 10.2021/trex.model. The datasets of human MEG data used during the current study are not available but are available from the corresponding author on reasonable request.

## Abbreviations

AD: Alzheimer’s disease
EEG: Electroencephalography
E/I: Excitation-inhibition
EPSP: Excitatory postsynaptic potential
fMRI: Functional magnetic resonance imaging
iPSC: Induced pluripotent stem cell
IPSP: Inhibitory postsynaptic potential
MCI: Mild cognitive impairment
MEG: Magnetoencephalography
MMSE: Mini-Mental State Examination
NMM: Neural mass model
