# Supplementary material for: A multiscale brain network model links Alzheimer’s disease-mediated neuronal hyperactivity to large-scale oscillatory slowing
Source: Alzheimers Res Ther. 2022 Jul 25;14:101. doi: 10.1186/s13195-022-01041-4 (PMC9310500; doi:10.1186/s13195-022-01041-4)
Supplement: Supplementary file 1 — Additional file 1: The neural mass model. Single neural mass analyses. Parameter range. Table S1. Single neural mass model. Fig. S1. Example traces of simulated MEG signals in control condition and several AD-like (and their contrast) scenarios. Fig. S2. The excitatory and inhibitory post-synaptic potentials (EPSP and IPSP). Fig. S3. Neuronal activity and total power in AD-like and contrast scenarios in a single, uncoupled, neural mass model. Fig. S4. Simulated power spectra for AD-like and contrast scenarios in a single, not-coupled, neural mass model. Fig. S5. Robust changes in outcome measures over a range of values for each relevant model parameter. [file 13195_2022_1041_MOESM1_ESM.docx]

**9. Supplementary information**

**A multiscale brain network model links Alzheimer’s disease-mediated neuronal hyperactivity to large-scale oscillatory slowing**

**9.1 The neural mass model**

The neural mass model used in this study consists of two populations: the excitatory and the inhibitory population. Both these neuronal populations are characterized by a membrane potential, described by the model as V_e_(t) and V_i_(t). The impulse responses reflect the postsynaptic activity and can change the membrane potentials of the neuronal populations. The following impulse responses were adapted from Zetterberg *et al*. (1978);

$$h\left( \tau\right)=A\left[ \exp\left( -a\tau\right)-\exp\left( -b\tau\right) \right] \mathrm{for} \tau\geq0$$

$$h\left( \tau\right)=0 \mathrm{for}\tau<0$$

Once a neuronal population receives an impulse, the average membrane potential is changed and the sigmoid functions S1(x) and S2(x) of the transfer altered membrane potentials to pulse density (or spike density). We used the S function also described by Zetterberg *et al.* (1987):

$$S\left[ Vm-Vd \right]=g\exp\left\{ q\left( Vm-Vd \right) \right\}\mathrm{for} Vm\leq Vd$$

$$S\left[ Vm-Vd \right]=g\left[ 2-\exp\left\{ q\left( Vd-Vm \right) \right\} \right]\mathrm{for} Vm>Vd$$

The parameter values can be found in table 1 of the main text.

C1 and C2 describe the strength of coupling between the two neuronal populations within the neural mass. The model is running by P(t) which is the pulse density representing the thalamic (excitatory) input to a excitatory population of a neuronal mass. Neural masses are coupled via excitatory connections with a fixed delay (T) and strength (S).

**9.2 Single neural mass analyses.**

To identify the local effects of AD-like mechanisms, spectral activity and spike density of 78 single (uncoupled) neural masses (Fig. S3 and S4) are analyzed. An overview of the results can be found in table S1. All AD-like mechanisms showed an increase in pyramidal neuronal spike density and total power, similar to AD-like lesions in the coupled neural mass network. However, AD-like pathology in single neural masses did not result in oscillatory slowing, and thus does not recapitulate whole-brain MEG data of human AD patients. On the contrary, the contrast scenarios showed a lower pyramidal neuronal spike density and total power compared to the control condition, and recapitulated oscillatory slowing as in human patients.

**Table S1. Single neural mass model**

|  | Hu-man  AD | AD-like scenarios | | | | | | Contrast scenarios | | | | | |
| --- | --- | --- | --- | --- | --- | --- | --- | --- | --- | --- | --- | --- | --- |
|  |  | 1A | 1B | 1C | 2A | 2B | 2C | 1A | 1B | 1C | 2A | 2B | 2C |
| Parameter |  | Vd1 | EPSP | S | Vd2 | IPSP | C2 | Vd1 | EPSP | S | Vd2 | IPSP | C2 |
| Direction |  | up | up | up | down | down | down | down | down | down | up | up | up |
| Oscillatory behavior | Slowing | Faster | Slowing | n.a. | Faster | Faster | Faster | Slowing | Slowing | n.a. | Slowing | Slowing | Slowing |
| Neuronal activity | n.a. | Higher | Lower | n.a. | Higher | Higher | Higher | Lower | No change | n.a. | Lower | Lower | Lower |
| Total power | Higher (n.s.) | Higher | Higher | n.a. | Higher | Higher | Higher | Lower | Lower | n.a. | Lower | Lower | Lower |

This table shows the direction of change in summarized MEG outcome measures for human MCI and simulated early AD-like or its opposite (contrast) scenarios compared to a (healthy) control conditions. Neuronal activity (*i.e.* spike density of pyramidal neurons) can only be assessed in model data. Of note, the interpretation of the indicated direction (up/down) of parameters are intuitive, such that ‘up’ of means, for instance, stronger coupling or hyperexcitability (which is actually derived by lowering the threshold potential). N.a. = not available. N.s. = not significant. AD = Alzheimer’s Disease.

**9.3 Parameter range**

We analyzed spectral power and spike density levels over a range of parameter values to investigate the robustness of our results (Fig. S5.). We show that, in general, the effect of AD-like pathology on simulated brain oscillations is similar for different strength of parameter changes. In contrast scenarios, the direction of change in outcome measure is less robust and non-linear, in particular in scenarios that also show a very strong decrease in total power. However, more extreme values for the different parameters do often generate unreliable physiological activity and are thus considered less relevant.


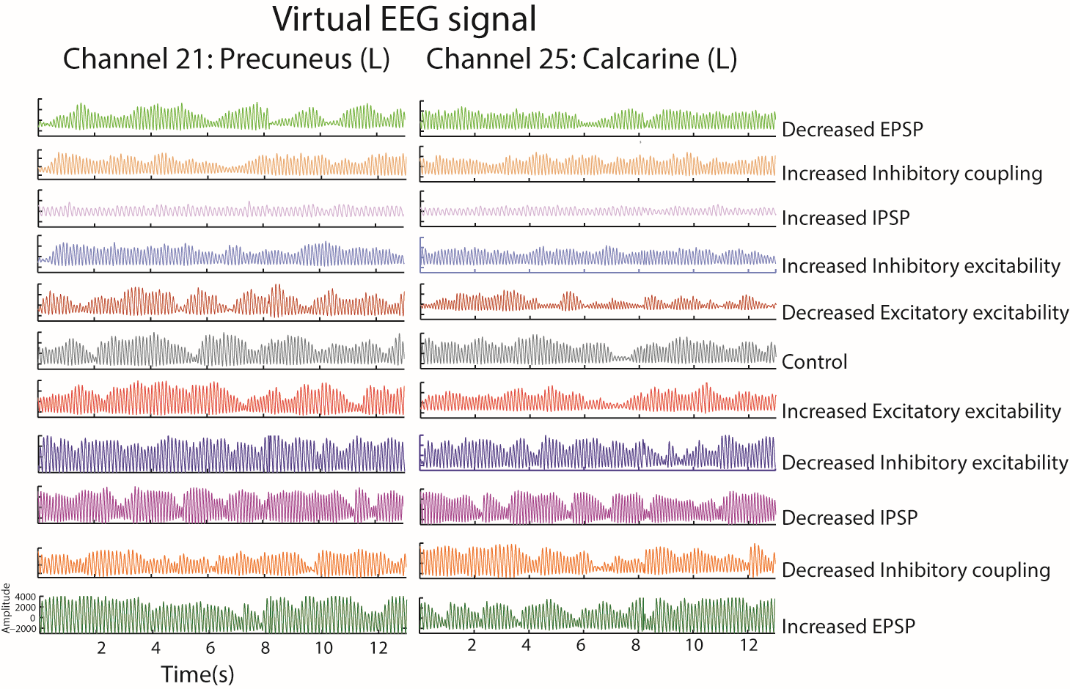


**Fig. S1. Example traces of simulated MEG signals in control condition and several AD-like (and their contrast) scenarios.** The two columns represent simulated neurophysiological signal from two different neural masses in a network of neural mass models that is coupled according to the Gong atlas. We show virtual activity of the precuneus and calcarine culcus of the left hemisphere. Each row represents a different scenario, either of AD-mediated neuronal dysfunction or its contrast scenario. The signals find their origin in fluctuations of the average membrane potential of the main pyramidal neuronal population over time.


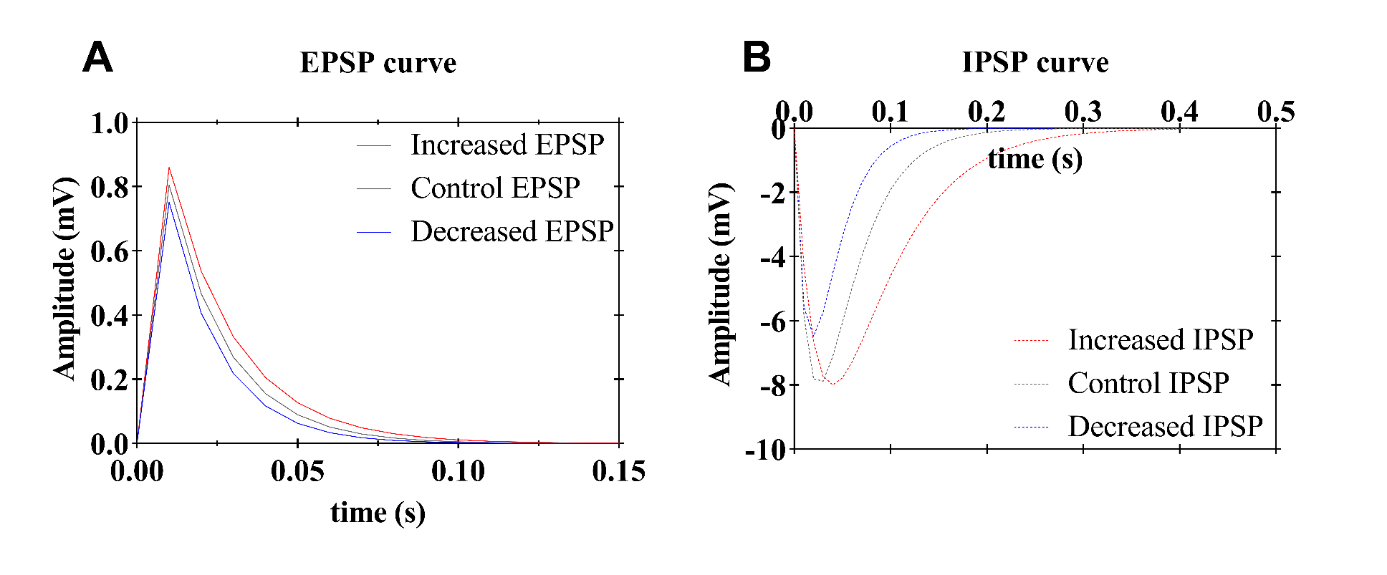
 **Fig. S2. The excitatory and inhibitory post-synaptic potentials (EPSP and IPSP).** The impulse response functions h_e_(t) and h_i_(t) determine the shape of the EPSP (A) and IPSP (B). In grey we show the EPSP (solid line) and IPSP (dashed line) in the ‘healthy’ control condition, in blue we show decreased (amplitude and duration) IPSP and EPSP and in red we show increased IPSP and EPSP.


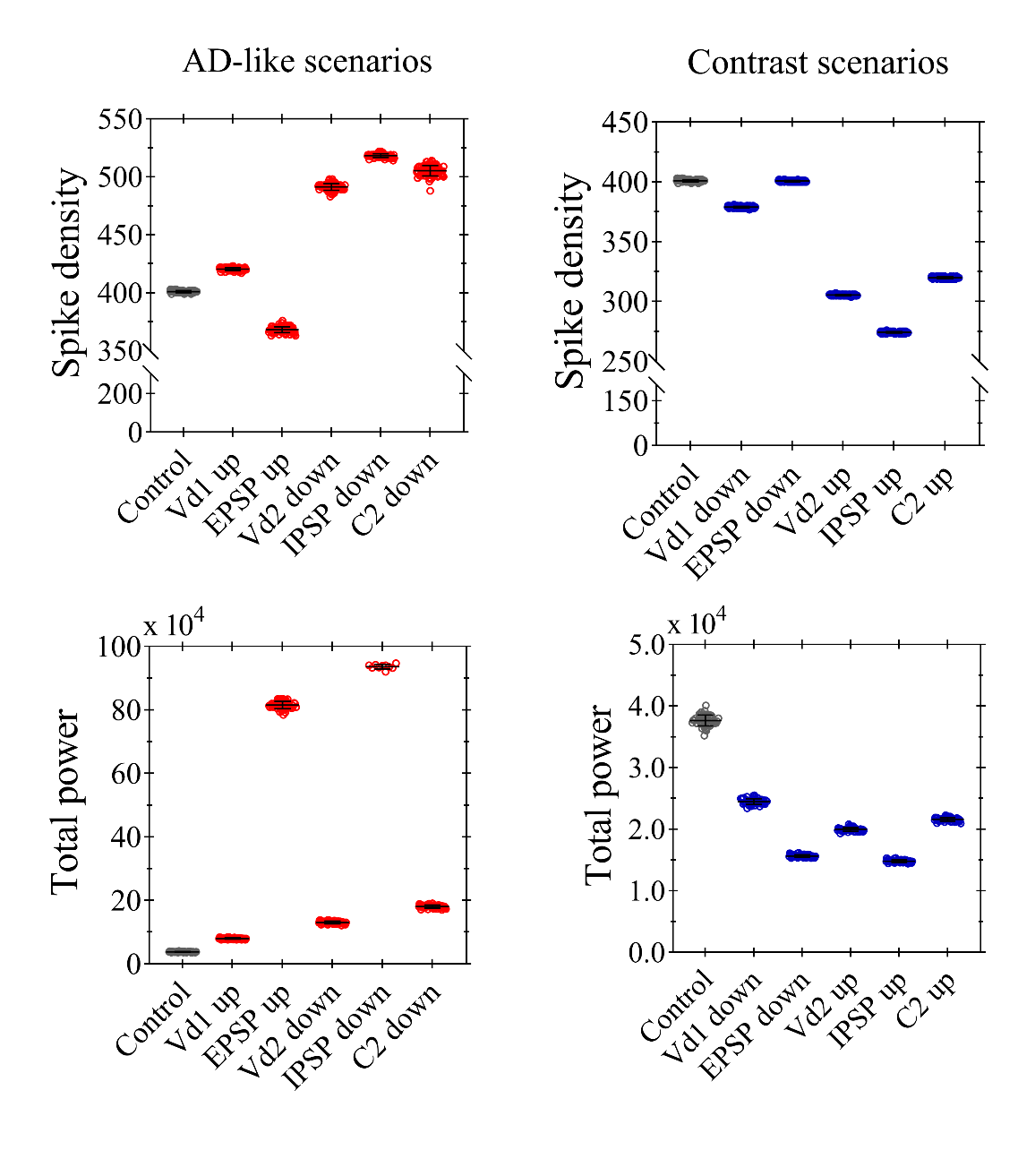


**Fig. S3. Neuronal activity and total power in** **AD-like and contrast scenarios in a single, uncoupled, neural mass model.** Single neural mass activity was investigated for AD-like (in red) and contrast scenarios (in blue) and compared to control conditions (in grey). Spike density is higher in the majority of the AD-like scenarios, while this is lower in all but one of the contrast scenarios. Total power is increased in all AD-like scenarios and decreased in all contrast scenarios.


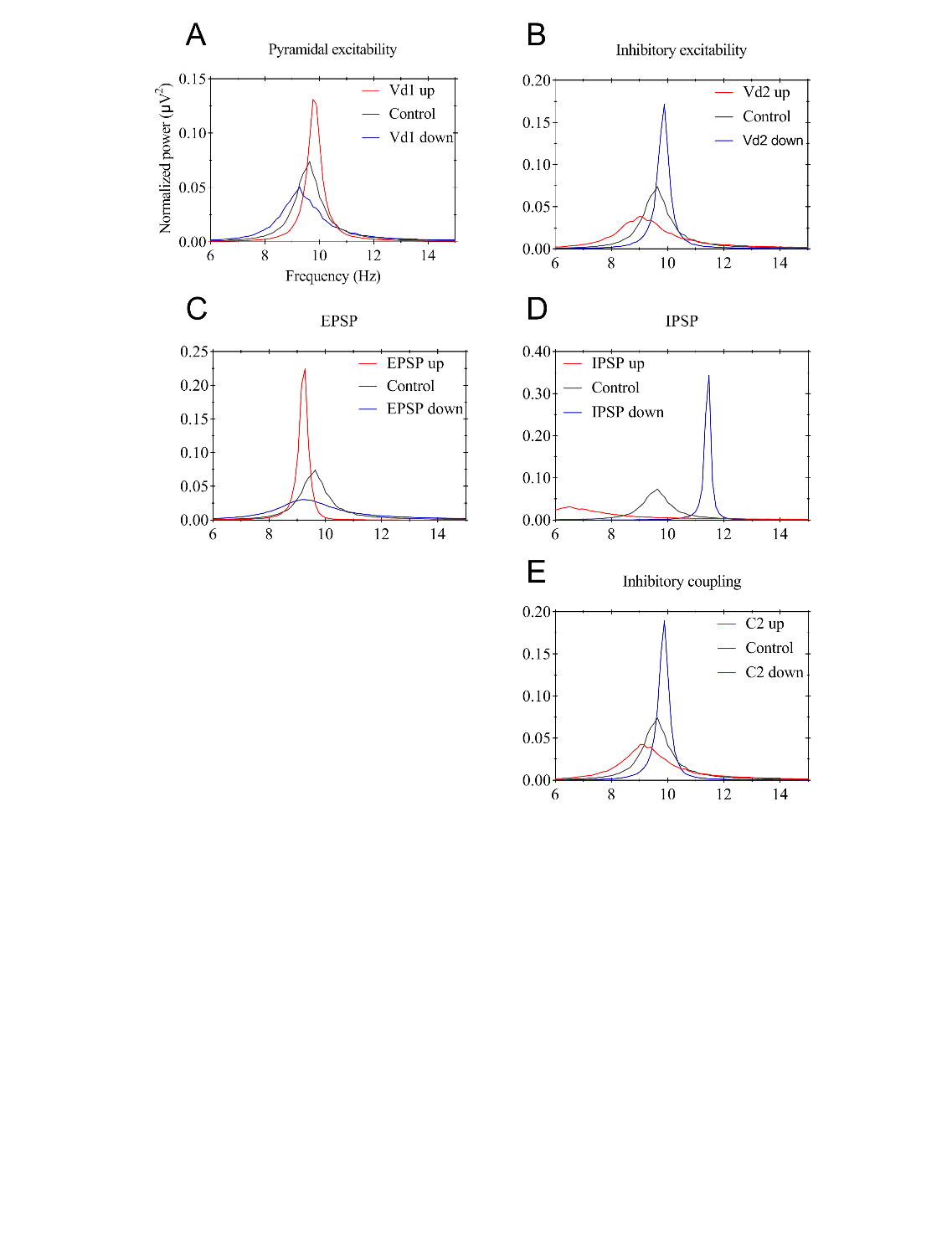
 **Fig. S4. Simulated power spectra for AD-like and contrast scenarios in a single, not-coupled, neural mass model.** (A) Increased pyramidal neuron excitability causes a shift in power spectrum to faster frequencies, while a decrease in excitability shifts the power to more slow frequencies. (B) Increased inhibitory neuron excitability causes a shift in power to more slower frequencies, while a decrease in excitability shifts the power to faster frequencies. (C) Increased excitatory post-synaptic potential (EPSP) in both pyramidal en inhibitory neurons results in a shift in power to more slow frequencies, while decreasing the EPSP also results in a slowing of the oscillatory activity. (D) Increased inhibitory post-synaptic potential (IPSP) of the pyramidal populations shows a shift in power spectrum to slower frequencies, while decreasing the IPSP increases the power in faster oscillations. (E) Increased inhibitory to excitatory coupling causes the power spectrum to shift to the left, i.e. to more slow frequencies, and decreased coupling results in a shift of power to faster oscillations compared to the control condition.


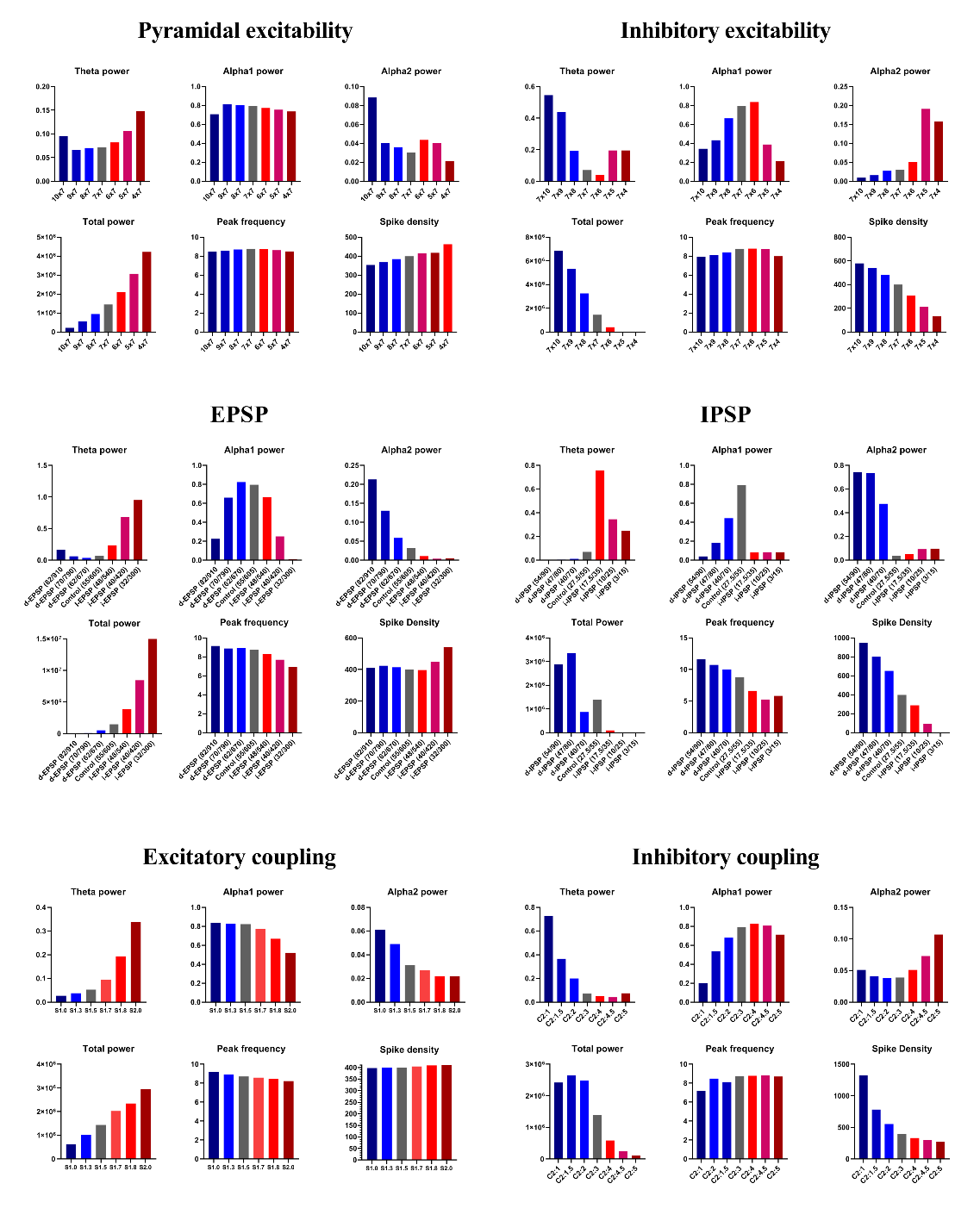


**Fig. S5. Robust changes in outcome measures over a range of values for each relevant model parameter.** This figure shows the outcome measures (total (absolute broadband) power, peak frequency (between 6 and 13Hz), spike density of the pyramidal neuron populations, relative power in theta, alpha 1 and alpha 2 frequency bands) for each simulation by the coupled dynamic brain network model. In grey we show the values for the control condition, reflecting a ‘healthy’ model. For each relevant model parameter (Excitatory/inhibitory excitability, postsynaptic impulse response or coupling) we either increased (in red) or decreased (in blue) the value to show what happens with the spectral activity of the simulated oscillations over a range of parameter values.
